# Global, regional and national epidemiology and prevalence of child stunting, wasting and underweight in low- and middle-income countries, 2006–2018

**DOI:** 10.1038/s41598-021-84302-w

**Published:** 2021-03-04

**Authors:** Paddy Ssentongo, Anna E. Ssentongo, Djibril M. Ba, Jessica E. Ericson, Muzi Na, Xiang Gao, Claudio Fronterre, Vernon M. Chinchilli, Steven J. Schiff

**Affiliations:** 1 Center for Neural Engineering, The Pennsylvania State University, University Park, PA 16802 USA; 2 Department of Engineering Science and Mechanics, The Pennsylvania State University, University Park, PA 16802 USA; 3 Department of Public Health Sciences, The Pennsylvania State University College of Medicine, 500 University Drive, Hershey, PA 17033 USA; 4 Department of Surgery, The Pennsylvania State University College of Medicine, Hershey, PA 17033 USA; 5 Center for Applied Studies in Health Economics, The Pennsylvania State University College of Medicine, Hershey, PA 17033 USA; 6 Department of Pediatrics, The Pennsylvania State University College of Medicine, Hershey, PA 17033 USA; 7 Department of Nutritional Sciences, College of Health and Human Development, The Pennsylvania State University, University Park, PA 16802 USA; 8 Centre for Health Informatics, Computing, and Statistics, Lancaster University, Lancaster, UK; 9 The Center for Infectious Disease Dynamics, The Pennsylvania State University, University Park, PA 16802 USA; 10 Department of Neurosurgery, The Pennsylvania State University College of Medicine, Hershey, PA 17033 USA; 11 Department of Physics, The Pennsylvania State University, University Park, PA 16802 USA

**Keywords:** Infectious diseases, Nutrition disorders

## Abstract

In 2016, undernutrition, as manifested in childhood stunting, wasting, and underweight were estimated to cause over 1.0 million deaths, 3.9% of years of life lost, and 3.8% of disability-adjusted life years globally. The objective of this study is to estimate the prevalence of undernutrition in low- and middle-income countries (LMICs) using the 2006–2018 cross-sectional nationally representative demographic and health surveys (DHS) data and to explore the sources of regional variations. Anthropometric measurements of children 0–59 months of age from DHS in 62 LMICs worldwide were used. Complete information was available for height-for-age (n = 624,734), weight-for-height (n = 625,230) and weight-for-age (n = 626,130). Random-effects models were fit to estimate the pooled prevalence of stunting, wasting, and underweight. Sources of heterogeneity in the prevalence estimates were explored through subgroup meta-analyses and meta-regression using generalized linear mixed-effects models. Human development index (a country-specific composite index based on life expectancy, literacy, access to education and per capita gross domestic product) and the United Nations region were explored as potential sources of variation in undernutrition. The overall prevalence was 29.1% (95% CI 26.7%, 31.6%) for stunting, 6.3% (95% CI 4.6%, 8.2%) for wasting, and 13.7% (95% CI 10.9%, 16.9%) for underweight. Subgroup analyses suggested that Western Africa, Southern Asia, and Southeastern Asia had a substantially higher estimated prevalence of undernutrition than global average estimates. In multivariable meta-regression, a combination of human development index and United Nations region (a proxy for geographical variation) explained 54%, 56%, and 66% of the variation in stunting, wasting, and underweight prevalence, respectively. Our findings demonstrate that regional, subregional, and country disparities in undernutrition remain, and the residual gaps to close towards achieving the second sustainable development goal—ending undernutrition by 2030.

## Introduction

In 2016, undernutrition (stunting, wasting and underweight) was estimated to cause 1.0 million deaths, 3.9% of years of life lost, and 3.8% of disability-adjusted life years (DALYs) globally^1^. Since then, undernutrition has decreased globally but remains endemic in southeastern Asia (SA) and sub-Saharan Africa (SSA)^2,3^. Importantly, heterogeneity still exists in the trends of undernutrition, with Africa being the only region where the number of stunted children continues to rise, from 50 million in 2000 to 59 million in 2018^4^.

Early childhood is a critical window during which significant growth and development occur. The nutrition of the mother and the early life nutrition of the child have a substantial impact on the child’s future physical and mental health. Undernutrition during this period is related to poor outcomes in overall health, neurobehavioral and cognitive development, and educational and economic attainment later in life^5,6^. Therefore, exploring country-level heterogeneity within ‘hot spots’ of child undernutrition is crucial to guide efforts to develop informed and focused control and prevention strategies.

Nutrition status is primarily assessed through the measurement of a child's height (or length in the youngest children) and weight and comparing the child to the standard metrics. Stunting (height-for-age z-score below − 2 standard deviations (SD) from the global median, as defined by the 2006 World Health Organization Child Growth Standards), wasting (weight-for-height z-score below − 2 SD from the global median) and underweight (weight-for-age z-score below − 2 SD from the global median) are indicators of a child’s undernutrition^7^. These anthropometric measures on a country level are updated regularly through the demographic and health surveys (DHS) program, which collects nationally representative health data to monitor and evaluate population health and nutrition programs in low- and middle-income countries (LMICs)^8^. Stunting, wasting and underweight have been assessed in SSA^3^, and stunting and wasting estimates are regularly updated by the United Nations’ Food and Agriculture Organization reports. However, we are unaware of prior studies of the combined influence of human development index (a country-specific composite index based on life expectancy, literacy, access to education and per capita gross domestic product) on all three undernutrition forms, at the global, regional and country-level.

To address this gap, we conducted a pooled analysis of stunting, wasting and underweight prevalence and explored the sources of undernutrition heterogeneity. We used country-level data on the prevalence of undernutrition from the DHS for the past twelve years (2006–2018). We limited our estimates to 2006–2018 due to the introduction of the World Health Organization (WHO) child growth standards in 2006, which estimated new values for assessing child nutritional status. These standards replaced the National Center for Health Statistics/World Health Organization (NCHS/WHO) growth reference, which had been in international use since the late 1970s but underestimated the prevalence of undernutrition, especially for infants.

The present study focuses on the spatial distribution of childhood stunting, wasting and underweight prevalence in 62 LMICs. Global, regional and country-specific information on the prevalence of the three forms of undernutrition will help guide policymakers, national and international efforts to control and prevent factors that drive undernutrition.

## Materials and methods

### Sampling process of demographic and health surveys

In this study, nationally representative DHS data between 2006 and 2018, including anthropometric indices for each country, were extracted^9^. Surveys without anthropometric data were excluded from the analysis (Supplementary Figure S1). The DHS Program collects nationally representative health data in LMICs every 5 years to monitor and evaluate population health and nutrition programs. The survey designs are based on stratified multistage sampling designs where each country is divided into administrative regions^10^. Populations within these regions are then stratified further by urban and rural areas of residence. The definition of urban or rural varies across countries. A random selection of enumeration areas or primary sampling units (PSUs) are drawn within rural or urban regions. PSUs are selected based on a probability proportional to the population size using the latest census. In most countries, a PSU is equivalent to the lowest administrative geographical unit such as a village. In the second sampling stage, all households within a PSU are listed from the most recent population census, and ~ 30 households per PSU are randomly selected for an interview. For each sampled household, all members are listed and have an equal chance of being sampled. Children between the ages of 0 and 59 months are eligible for anthropometric measurements, and had their heights (or lengths) and weights measured by trained field-workers.

### Anthropometry methods and assessment of undernutrition status

Children of age 24 months and younger had their length measured in recumbent position but for ages above two years had their height measured while standing. Length was measured with the portable Harpenden Infantometer (range 30–110 cm, with digit counter readings precise to 1 mm), and the height with the Harpenden Portable Stadiometer (range 65–206 cm, digit counter reading). Portable electronic scales with a taring capability, calibrated to 0.1 kg, were used to measure weight^11,12^. The z-scores for weight-for-age, weight-for-height, and height-for-age were provided in the DHS data and were calculated using the 2006 WHO Child Growth Standards. The 2006 WHO Child Growth Standards replaced the NCHS/WHO growth reference curves, which had been in used as an international growth reference since 1977^13,14^. Unlike the NCHS/WHO growth reference, which is based on children from a single country, the WHO standards depict normal early childhood growth under optimal environmental conditions and can be used to assess children everywhere, regardless of ethnicity, socioeconomic status and type of feeding^15^. Another difference lies in the methodology applied to construct the growth curves. The computation of growth curves and the z-scores for the new WHO standards uses formulae based on the LMS method^16^. For these reasons, the DHS conducted after 2006 that used the new growth standards were analyzed. A child was stunted, wasted, or underweight if he or she exhibited a *z* score below − 2. The present study is a secondary data analysis. Country-specific number of children with anthropometric measurements are reported in Supplementary Table S1.

### Human development index (HDI)

To assess sources of variability in the prevalence of undernutrition in LMICs, we fitted our generalized linear mixed-effects models with the 2018 HDI (Supplementary Figure S2)^17^. The HDI is the geometric mean of normalized indices for each of the three human development measures: education, life expectancy and economy. The education dimension is measured by average years of schooling for adults 25 years and older and expected years of schooling for children entering school age. The economic dimension is measured by gross national income per capita, and the health dimension is assessed by life expectancy at birth. The HDI ranges from 0 to 1, with a higher score indicative of higher HDI. The official categorization by the United Nations is low, medium, high and very high. Educational attainment for women of reproductive age is one of the leading social determinants of health, with higher attainment associated with improved child nutritional outcomes in LMICs^18,19^.

### Study selection and inclusion criteria

All countries with complete DHS anthropometric information between 2006 and 2018 were included in the analysis. The following variables were extracted: year of the survey, continent, United Nations (UN) region, UN subregions (Supplementary Figure S3), number of children stunted, number of children wasted, number of children underweight, and the total population of children whose weight and lengths/heights were measured. We excluded surveys that were not within the time frame of (2006–2018) or did not report anthropometric indicators of children under 5 years.

### Statistical analysis

The primary outcome was an estimation of the prevalence of stunting, wasting and underweight. The DHS program reported undernutrition prevalence based on the total number of children with completed anthropometric measurements; therefore, we reported the prevalence as a percent of the children under 5 years with anthropometric measurements.

We applied random-effects models to estimate the overall global prevalence of undernutrition and their respective 95% confidence intervals (CIs) using DerSimonian and Laird random-effects meta-analytic method (See equations in supplementary material)^20^. A random-effects model assumes the observed prevalence estimates can vary across surveys because of real differences in the measured effect that are independent of time and represent unmodeled variance. To pool the estimates, we built random-effects models using the inverse variance method with logit transformed proportions^21,22^. Individual and pooled estimates were graphically displayed via forest plots. Between-study variation (heterogeneity) was assessed using ${I}^{2}$, which describes the percentage of total variation across surveys that is due to heterogeneity rather than chance, expressed as percent (low (25%), moderate (50%), and high (75%)^23^.

A generalized linear mixed-effects meta-regression model with a logit link function was fit to investigate the sources of heterogeneity and the results were reported as odds ratios (OR) and their corresponding 95% confidence intervals (95% CI). We examined the associations of each of the explanatory variables included in the model in relation to undernutrition prevalence. These included country-level HDI and the subregions classified by the United Nations^24^. The *metaprop*, *escalc*, *rma* functions from the R packages *meta* and *metafor* were used for the analysis^25^.

### Ethics approval and consent to participate

The DHS program takes strict measures for protecting the privacy of all survey respondents. Procedures and questionnaires for standard DHS surveys have been reviewed and approved by the ICF International Institutional Review Board (IRB) and the IRB of the host country. ICF International provides both writing and oral informed consent to each survey respondent before the beginning of each survey question and biomarker tests. Each participant's participation was voluntary. This study protocol was submitted to the Pennsylvania State University institutional review board and was not considered to be human subject research, as defined by the US Department of Health and Human Services.

### Consent for publication

No consent to publish was needed for this study as we did not use any details, images or videos related to individual participants.

## Results

We identified 93 potentially relevant DHS from LMICs. Thirty-one surveys were excluded because they were conducted before 2006 or had no data on anthropometric measures. The remaining 62 surveys, each representing one country, provided data for this pooled analysis. Thirty-eight countries were from Africa, 14 from Asia and 8 from Latin America and the Caribbean, and 1 each from Oceania and Europe. Supplemental Table S1 shows the characteristics of the 62 surveys that we included in the analyses.

**Figure 1:**
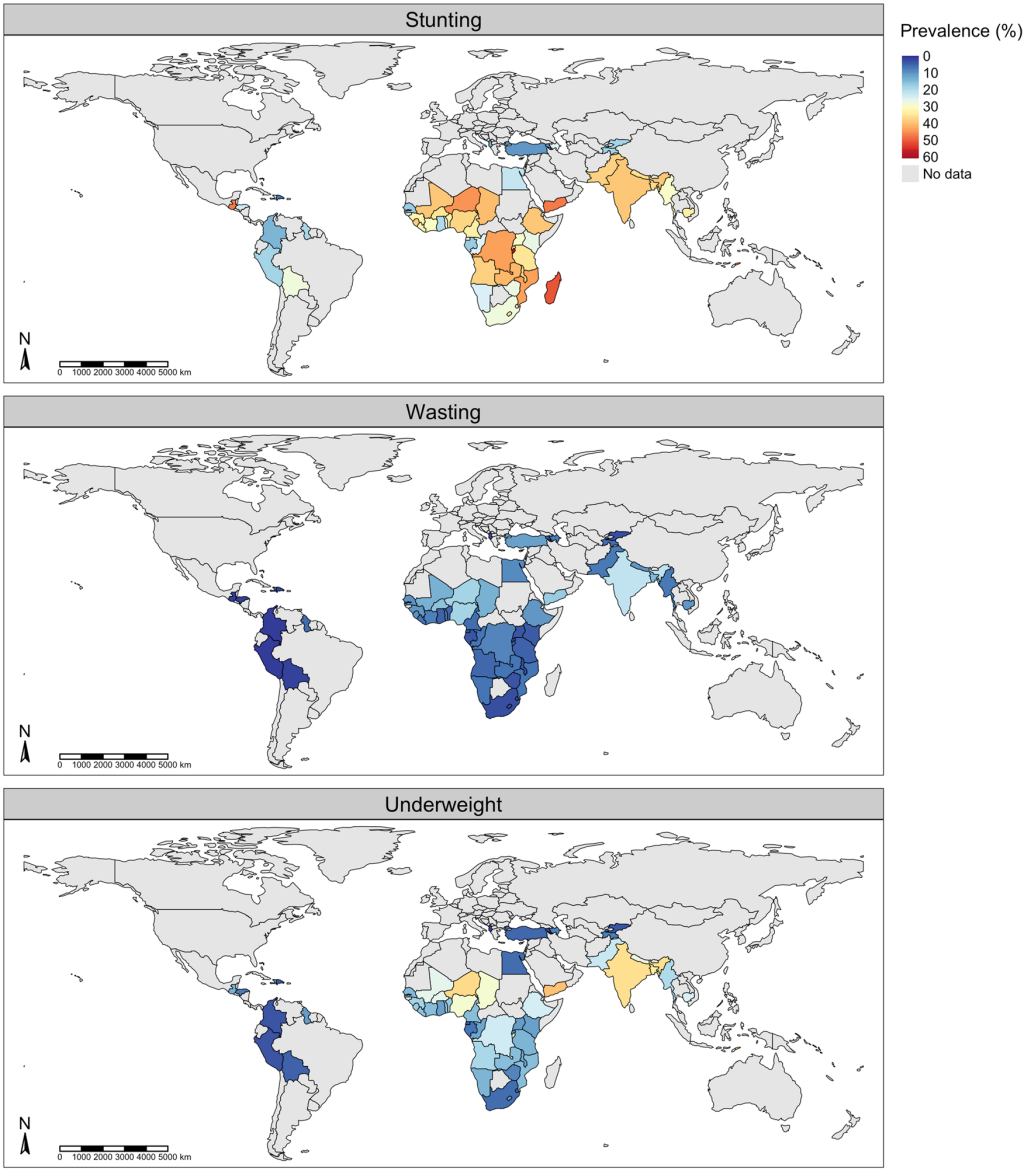
Prevalence of undernutrition. Countries are shaded according to prevalence (%) of stunting (top row), wasting (middle row) and underweight (bottom row).

The overall prevalence was 29.1% (95% CI 26.7%, 31.6%) for stunting, 6.3% (95% CI 4.6%, 8.2%) for wasting, and 13.7% (95% CI 10.9%, 16.9%) for underweight. Substantial heterogeneity was evident (*I*^2^ = 100%;* p* for heterogeneity < 0.0001). Figure 1 is a world map displaying country-level prevalence of stunting, wasting and underweight.

### Stunting

**Figure 2:**
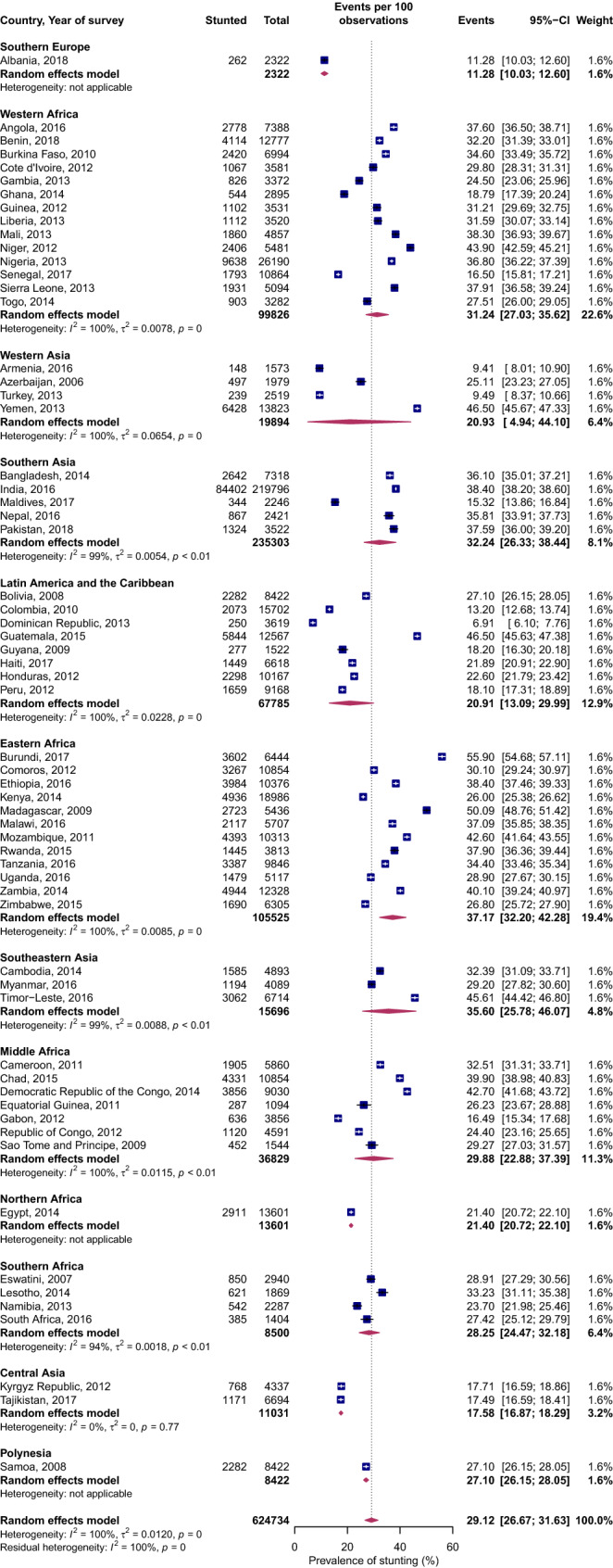
Forest plot of stunting prevalence by UN subregions of LMICs: event values represent the number of cases of stunting expressed as a percentage. Blue squares and their corresponding lines are the point estimates and 95% confidence intervals (95% CI). Maroon diamonds represent the pooled estimate of the prevalence for each subgroup (width denotes 95% CI). Weights are from the random-effects meta-analysis model described by DerSimonian and Laird^20^ (*p* for interaction comparing the different subgroups < 0.0001).

Figure 2 shows the prevalence of stunting among children under age five years on a subregional and country level. Overall, Africa (Supplemental Figure S4) had the highest prevalence of stunting at 32% (95% CI 29.5–35.9%), followed by Oceania 27% (95% CI 26.2–28.1%) and Asia at 27.4% (95% CI 22.1–32.0%). The Americas and Europe had the lowest prevalence of stunting: 20% (95% CI 13.1–29.1%) and 11.3% (95% CI 10.0–12.6%), respectively. The prevalence of stunting in Africa was significantly different from America, Europe and Oceania but not Asia.

Within the Africa region, the prevalence of stunting in Northern Africa was on average 10 percentage points less than that of Middle, Eastern, Western and Southern Africa. Within the SSA region, the prevalence of stunting in Southern Africa was less than half that of Middle, Eastern, and Western Africa. Similarly, the prevalence of stunting for Southern and Southeastern Asia was almost two times the burden of stunting in Western and Central Asia, and 10 percentage points greater than in Eastern Asia.

At the country level, nations with high stunting prevalence (all with prevalence greater than 40%) were mostly SSA countries—Burundi, Democratic Republic of Congo, Madagascar, Mozambique, Niger and Zambia. Yemen in Western Asia, Timor-Leste in Southeastern Asia and Guatemala in Southern America also displayed a stunting prevalence of 40% and higher. Several countries had high prevalence of both stunting and wasting. For example, Timor-Leste in Southeastern Asia and India in Southern Asia had high prevalence of both stunting and wasting.

### Wasting

**Figure 3:**
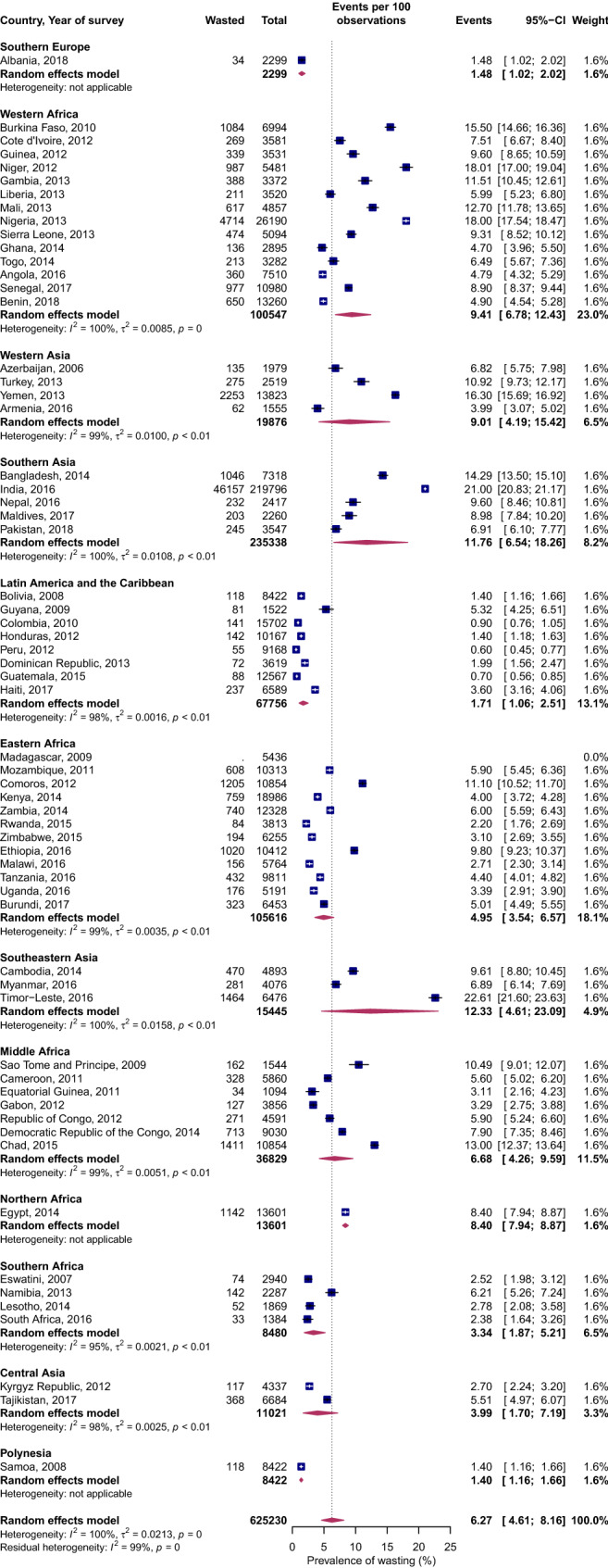
Forest plot of wasting prevalence by UN subregions of LMICs: event values represent the number of cases of wasting expressed as a percentage. Blue squares and their corresponding lines are the point estimates and 95% confidence intervals (95% CI). Maroon diamonds represent the pooled estimate of the prevalence for each subgroup (width denotes 95% CI). Weights are from the random-effects meta-analysis model described by DerSimonian and Laird^20^ (*p* for interaction comparing the different subgroups < 0.0001).

Figure 3 shows the prevalence of wasting on a subregional and country level. On a regional level (Supplemental Figure S5), the prevalence of wasting in Asia was 3 percentage points greater than that of Africa but 7 to 8 percentage points greater than that of Europe, the Americas and Oceania. On a subregional level, substantial variations in the burden of wasting existed. For example, in Africa, the prevalence in Western Africa was almost twice as high as that of Eastern and Middle Africa and three times that of Southern Africa. At the country level, 13 nations had > 10% prevalence of wasting. These included five countries from Asia—Timor-Leste, India, Bangladesh, Yemen and Turkey, and eight countries from SSA—Nigeria, Niger, Burkina Faso, Mali, Chad, Sao Tome Principe, The Gambia, and Comoros.

### Underweight

**Figure 4:**
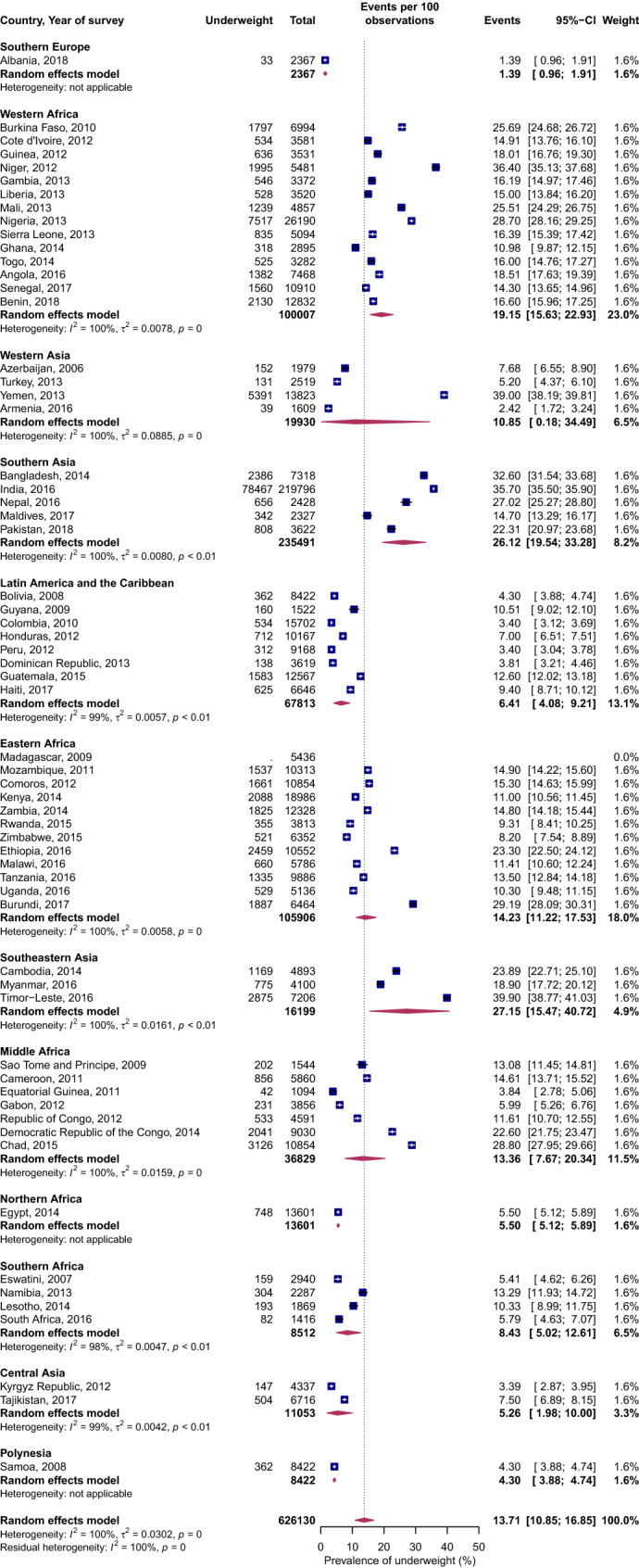
Forest plot of underweight prevalence by UN subregions of LMICs: event values represent the number of cases of underweight expressed as a percentage. Blue squares and their corresponding lines are the point estimates and 95% confidence intervals (95% CI). Maroon diamonds represent the pooled estimate of the prevalence for each subgroup (width denotes 95% CI). Weights are from the random-effects meta-analysis model described by DerSimonian and Laird^20^ (*p* for interaction comparing the different subgroups < 0.0001).

Figure 4 shows the prevalence of underweight on the subregional and country level. The prevalence of underweight in Africa and Asia was twice that of the Americas, four times that of Oceania and more than ten times that of Europe (Supplemental Figure S6). Within SSA, Western Africa had the highest prevalence of underweight, with its pooled estimates twice that of Southern Africa. However, Western African underweight prevalence was not substantially different compared to Eastern and Middle Africa. Similarly, the prevalence of underweight in Central Asia was, on average, five times less than that of Eastern, Southeastern and Southern Asia. Countries that displayed a very high prevalence of underweight (all with a prevalence ≥ 30%) were SSA countries, Niger and Burundi, and in Asia, India, Timor-Leste, Bangladesh and Yemen. On the contrary, the prevalence of underweight in Latin America and the Caribbean were, on average, substantially lower than the pooled overall prevalence.

### Association of human development index and UN region with stunting, wasting and underweight

**Figure 5:**
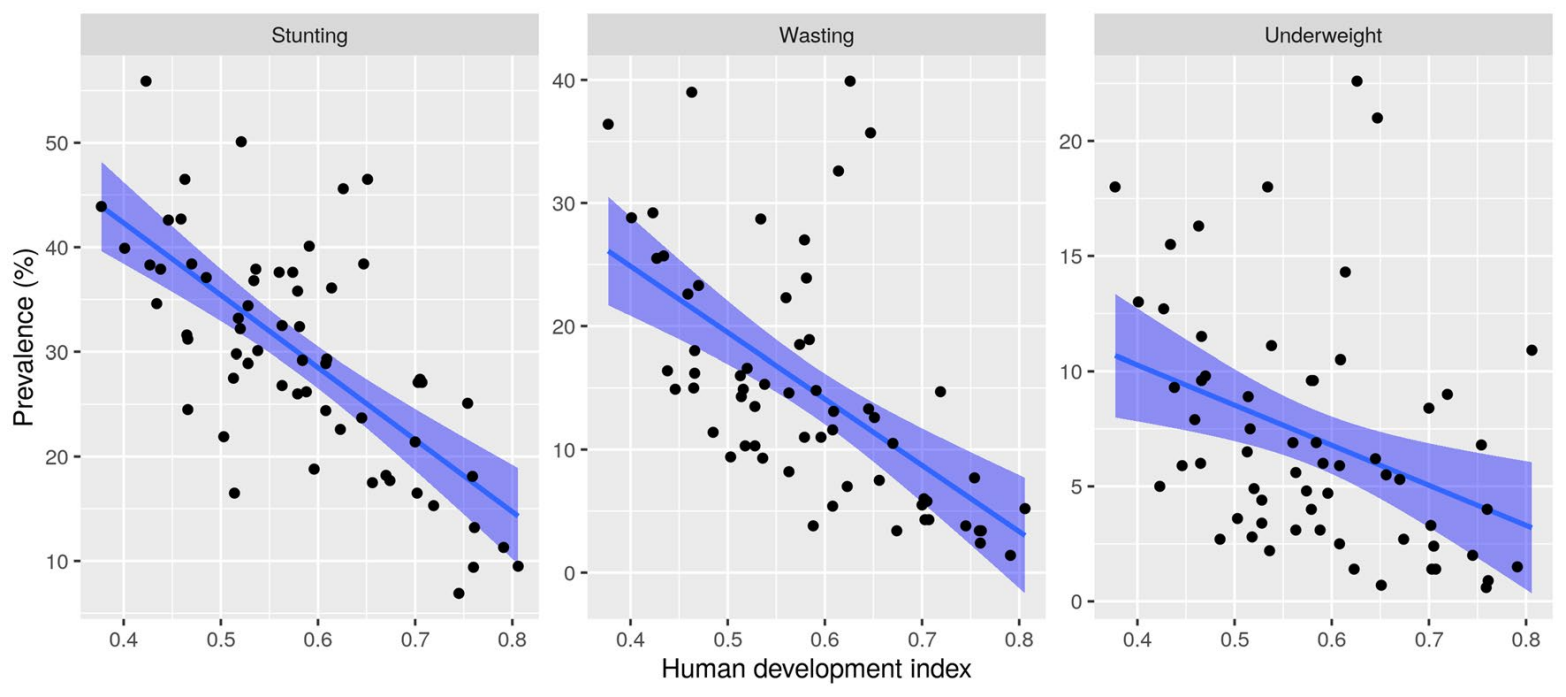
Correlation of HDI with undernutrition: A moderate negative correlation exists between HDI and stunting (Spearman’s rho; − 0.65, *p* value < 0.0001, first column), wasting (Spearman’s rho; − 0.43, *p* value = 0.0006, second column) and underweight (Spearman’s rho; − 0.67, *p* value < 0.0001, third column).

A moderate negative correlation was observed between the country’s HDI and all undernutrition forms (Fig. 5). Stunting (Spearman’s rho; − 0.65, *p* value < 0.0001), wasting (Spearman’s rho; − 0.43, *p* value = 0.0006) and underweight (Spearman’s rho; − 0.67, *p* value < 0.0001). The generalized linear mixed-effects meta-regression model suggested higher HDI was associated with lower odds of all three forms of undernutrition. For each increase in the level of HDI, the odds were 40%, 37%, and 51% lower for stunting, wasting, and underweight, respectively.

The United Nations region was associated with the prevalence of undernutrition. Eastern and Middle Africa, Southern and Southeastern Asia, and Polynesia displayed higher odds of stunting compared to Central Asia (Table 1). Wasting prevalence odds were significantly higher in Western, Southern, and Southeastern Asia, and Northern Africa than in Central Asia. However, Latin America and the Caribbean had lower odds of wasting compared to Central Asia. In the model, combining HDI and UN subregions (as a proxy for spatial variation) explained 54%, 56%, and 66% for stunting, wasting and underweight prevalence, respectively.

**Table 1:** Maximum likelihood estimates with associated 95% confidence intervals (CI) for the generalized linear mixed-effects model's regression coefficients.

Variable	Stunting		Wasting		Underweight	
	Odds ratio (95%CI)	*p* value	Odds ratio (95%CI)	*p* value	Odds ratio (95%CI)	*p* value
**UN subregion**						
*Africa*						
Eastern Africa	1.88 (1.06, 3.36)	0.032	0.87 (0.42, 1.81)	0.718	1.79 (0.95, 3.36)	0.073
Western Africa	1.36 (0.76, 2.43)	0.296	1.65 (0.80, 3.42)	0.176	2.35 (1.25, 4.40)	0.008
Middle Africa	1.82 (1.01, 3.29)	0.047	1.57 (0.75, 3.30)	0.235	2.39 (1.25, 4.55)	0.008
Southern Africa	1.84 (0.97, 3.48)	0.062	0.82 (0.36, 1.84)	0.629	1.65 (0.82, 3.31)	0.160
Northern Africa	2.11 (0.84, 5.30)	0.110	3.60 (1.14, 11.36)	0.029	2.21 (0.82, 6.00)	0.119
*Asia*						
Western Asia	1.44 (0.76, 2.75)	0.266	2.90 (1.29, 6.52)	0.010	2.53 (1.25, 5.12)	< .00001
Southern Asia	2.41 (1.30, 4.46)	0.005	3.49 (1.61, 7.58)	0.002	7.44 (3.80, 14.57)	< .00001
Southeastern Asia	2.57 (1.31, 5.04)	0.006	3.28 (1.41, 7.63)	0.006	6.82 (3.28, 14.15)	< .00001
Central Asia	Reference		Reference		Reference	
*Americas*						
Latin America and the Caribbean	1.41 (0.79, 2.54)	0.247	0.45 (0.22, 0.95)	0.036	1.56 (0.83, 2.96)	0.170
*Oceania*						
Polynesia	2.89 (1.15, 7.23)	0.024	0.56 (0.17, 1.78)	0.323	1.71 (0.63, 4.64)	0.295
*Europe*						
Southern Europe	0.99 (0.39, 2.49)	0.976	0.58 (0.18, 1.93)	0.376	0.53 (0.19, 1.52)	0.239
HDI	0.60 (0.50, 0.72)	< .00001	0.63 (0.50, 0.79)	< .00001	0.49 (0.40, 0.60)	< .00001

Odds ratios and their 95% confidence intervals for the association of United Nations regions and Human Development Index with undernutrition (stunting, wasting and underweight). The multivariable model included UN subregions and human development index. R^2^ is the coefficient of determination indicating the amount of variation explained by the variables. The R^2^ when HDI and UN subregions are included in the random-effects model is 54%, 56%, and 66% for stunting, wasting and underweight. Higher HDI was associated with lower odds of all three forms of undernutrition.

## Discussion

We estimated the prevalence of childhood (0–59 months) undernutrition (stunting, wasting and underweight) using data from the most recent DHS from 62 LMICs. Our results suggest that exploring undernutrition on a global or regional level could mask the unique differences of the disease burden within a sub-region level. Western Africa, Southern, and Southeastern Asia consistently displayed a substantial burden of stunting, wasting and underweight. Six of the nine countries with the highest burden of stunting were from SSA and two were from Asia. A combination of human development index and United Nations region (a proxy for geographical variation) explained 54%, 56%, and 66% of the variation in stunting, wasting, and underweight prevalence, respectively. The residual unexplained variance implied by these figures after optimizing for random effects suggest there are additional factors involved in these disparities.

The results of this study demonstrate a moderate association between a country's HDI with undernutrition. One of the components of HDI is the gross national income (GNI). Thus, countries with lower GNI had higher prevalence of undernutrition. Eleven percent of the world's population is living in poverty, defined by The World Bank as living on less than US$1·90 per day. Poverty disproportionally impacts children particularly those living in SSA and Southern Asia^26^. As a result, low- and middle-income countries have the highest burden of stunting, wasting and underweight and children in SSA and Southern Asia are disproportionately affected. The United Nation’s Millennium Development Goals include eradicating extreme poverty and hunger as the priority goal^27^, and continues to be a key global development agenda under the Sustainable Development Goals (SDGs)^28^. Like poverty, undernutrition often occurs in an intergenerational cycle. In addition to being a risk factor for infections and food insecurity—both of which are drivers of undernutrition—poverty is a pivotal contributor to allostatic load (the cumulative wear and tear on the body due to adapting to adverse physical or psychosocial stress), which modulates the biological mechanisms that influence growth^26^. Consequently, early childhood growth failure in LMICs has persisted despite decades of nutritional interventions. Children born into low-income families have, on average, poorer growth, poorer neurocognitive outcomes, and poorer educational attainment than wealthier peers. Such setbacks are, in turn, associated with lower economic productivity, thus contributing to the intergenerational transmission of poverty and undernutrition.

Lower HDI is associated with higher rates of infectious diseases^29–32^. The interaction between undernutrition and infection creates a lethal cycle of worsening illness and deteriorating nutritional status. In regions that are profoundly affected by undernourishment, infection prevalence rates are also high. SSA and South and Southeastern Asia are disproportionately affected by malaria, human immunodeficiency virus (HIV), and tuberculosis (TB), all of which are associated with worsening the nutritional status of the child^33^. Above and beyond the immediate outcomes such as death, infectious diseases expose children to a complex constellation of developmental risk factors embedded in contexts of biological stress and psychosocial disadvantage that perpetuates child growth failure. Infections induce a nutritional demand on biological systems through fevers and increased catabolic states by mediators of inflammation^34^. This results in increased energy expenditure so that fewer calories are available to support growth. Furthermore, infections often decrease food intake and alter digestion and absorption, further worsening the nutritional status. Undernutrition increases susceptibility to infection, and on the other hand, infections propagate deterioration of nutritional status, resulting in a synergistic cycle of undernutrition-infection that ultimately leads to severe undernutrition^35^.

### Public health relevance and recommendation

Adopted by the UN General Assembly in the September of 2015, the Sustainable Development Goals represent a new coherent way of thinking about how issues as diverse as ending poverty (goal 1), ending hunger (goal 2), health promotion (goal 3), achieving quality education for all (goal 4), achieving gender equality (goal 5), and climate change, fit together to foster international development^36^. Particularly, SDG 2.2 calls for an end to all forms of malnutrition by 2030, and is inseparable from many of the other SDG^37^. The updated quantitative assessments of levels of undernutrition indicators presented here should inform strategies at the regional and national levels targeted at achieving these SDGs. Lastly, the substantial unexplained variance in our findings emphasizes the need for further characterization of the correlates of undernutrition in LIMCs including genomics, gut microbiota, ethnicity, diet composition, micronutrients, climate change and weather variability^38–43^. Such analysis should also focus on using more granular subnational data.

### Strengths and limitations

The strengths of our study include that we only analyzed anthropometric measurement using the new modified 2006 WHO international standards of child growth measurements^7^. The nutrition analyses done using older growth references do not adequately represent early childhood growth^44,45^. In addition, we analyzed all three indicators of undernutrition at the global, regional, and subregional scale. These three indicators are distinct on their effect on the physical and neurocognitive outcome of the child, and therefore should be presented separately^46^.

Our study had some limitations. First, because we employed an ecological (aggregated data) study design, our findings may suffer bias and confounding from the ecological fallacy (drawing conclusions on individuals using aggregated data, even though the relevant individuals may not share such characteristics)^47^. Second, WHO international child growth standards data used were a global median. Because height is heritable there are likely genetic variations in height at the country-level not accounted for^48,49^. Nevertheless, at younger ages, differences in height and weight in preschool children that is explained by ethnic background are relatively small—3% for height and about 6% for weight, and the differences in these anthropometric measures at such ages are primarily driven by nutrition status of the child^50,51^. The WHO attempted to overcome this limitation by representing the major global geographic regions in generating new growth curves^11^. Thirdly, although we limited the analysis to the recent nationwide DHS data, the twelve-year window (2006–2018) is quite broad and the relative metrics of under nutrition continue to evolve with time. Finally, due to the bidirectional and complex relationship between major infectious diseases ( including malaria, HIV, TB) and undernutrition^52^, we did not include these diseases in the meta-regression analysis since we cannot establish a causal link especially with the cross-sectional nature of the present study. Future longitudinal studies of individual-level data should estimate the contribution of major endemic infectious diseases to the risk of childhood undernutrition in multiple LMICs.

## Conclusion

In summary, substantial regional, subregional and country-level disparities of stunting, wasting and underweight still exist. The updated quantitative assessments of levels of undernutrition indicators presented here should inform strategies at the global, regional and national levels targeted at further reducing the remaining substantially undernourished populations.

## Supplementary Information

Supplementary Information 1.

Supplementary Information 2.

## Data Availability

The analyzed dataset is freely available from: https://dhsprogram.com/data/available-datasets.cfm. R code and data to reproduce the results in the present manuscript are archived at https://github.com/Schiff-Lab/Global-Malnutrition-Burden-Scientific-Reports-2021.

## Abbreviations

LMICs: Low- and middle-income countries
WHO: World Health Organization
SSA: Sub-Saharan Africa
DHS: Demographic and health surveys
UN: United Nations
CI: Confidence Interval
GDP: Gross domestic product
HDI: Human development index
