# Supplementary material for: Global, regional and national epidemiology and prevalence of child stunting, wasting and underweight in low- and middle-income countries, 2006–2018
Source: Sci Rep. 2021 Mar 4;11:5204. doi: 10.1038/s41598-021-84302-w (PMC7933191; doi:10.1038/s41598-021-84302-w)
Supplement: Supplementary file 1 — Supplementary Information 1. [file 41598_2021_84302_MOESM1_ESM.zip › R code and Data/SciReports_Undernutrition.nb.html]

R Script to reroduce results of ;Global, regional and country epidemiology and prevalence of stunting, wasting and underweight in low- and middle- income countries, 2006-2018


Code 

- Show All Code
- Hide All Code
- Download Rmd

# R Script to reroduce results of ;Global, regional and country epidemiology and prevalence of stunting, wasting and underweight in low- and middle- income countries, 2006-2018

# clear R global environment

rm(list=ls())

#Load the libraries


```
# load libraries
library(olsrr) # linear regression
library(tidyverse) # general stuff
library(hrbrthemes) # pretty plots
library(ggplot2) # pretty plots
library(plotly) # interactive plots
library(gapminder) # interactive plots
library(pastecs) # stat.desc
library(metafor) # for meta-analysis: mixed-effects logistic and Poisson regression models
library(meta) # meta-regression, GLMM, forest plots,(subgroup) meta-analyses.
library(sp) # spatial data
library(rgdal) #  projection/transformation operations for shapefiles
library(sf) # Simple Features for R
library(rnaturalearth)
library(tmap) # cool maps
library(ggpubr) # 'ggplot2' Based Publication Ready Plots
library(ggpmisc) #Miscellaneous Extensions to 'ggplot2'
library(spData)
library(cowplot)# plot grid
```

# load data

world


```
dat <- read.csv(file="DHS77_META.csv", header=T,stringsAsFactors=F)
hdi<- read.csv(file="HDI.csv", header=T,stringsAsFactors=F)
educ<-read.csv(file="Women_Education.csv")
dat <- merge(dat,hdi, by=c("Country"), all.x=T)
dat <- merge(dat,educ, by=c("Country"), all.x=T)
#dat_meta <- merge(dat,hdi, by=c("Country"), all.x=T)
dat$name=dat$Country
```

# display maps


```
WorldData <- map_data('world')
WorldData %>% filter(region != "Antarctica") -> WorldData
WorldData <- fortify(WorldData)
p <- ggplot()
p <- p + geom_map(data=WorldData, map=WorldData,
                  aes(x=long, y=lat, group=group, map_id=region),
                  fill="white", colour="#7f7f7f", size=0.5)
```


```
Ignoring unknown aesthetics: x, y
```


```
p <- p + geom_map(data=dat, map=WorldData,
                  aes(fill=stunt, map_id=Country),
                  colour="#7f7f7f", size=0.5)
p <- p + coord_map("rectangular", lat0=0, xlim=c(-180,180), ylim=c(-60, 90))
#p <- p + scale_fill_continuous(low="thistle2", high="darkred", guide="colorbar")
p <-p+ scale_fill_distiller(palette = "Spectral")
p <- p + scale_y_continuous(breaks=c())
p <- p + scale_x_continuous(breaks=c())
p <- p + labs(fill="Stunting(%)", title="", x="", y="")
p <- p + theme_bw()
p <- p + theme(panel.border = element_blank())
par(mai=c(0,0,0.2,0),xaxs="i",yaxs="i")
p
```


```
world_stunt = world %>%
  filter(continent!= "Antarctica", !is.na(iso_a2)) %>%
  #filter(continent == "Africa", !is.na(iso_a2)) #%>%
  left_join(worldbank_df, by = "iso_a2") %>%
  dplyr::select(name, subregion, gdpPercap, HDI, pop_growth) %>%
  left_join(dat, by = "name") %>%
  st_transform("+proj=longlat +datum=WGS84 +no_defs")
worldbank_df
```


```
# Map the output
tm_shape(world_stunt) + tm_polygons(col="HDI_2018", style="quantile") +
  tm_legend(outside=TRUE)
```

# stunting meta-analysis: Figure 1


```
# Fit model
m1.pft <- metaprop(stunt_n, Height_Age_N, data = dat, studlab = paste (Country, Year, sep = ", "), comb.fixed =F,
                   sm = "PFT", pscale = 100, method.ci = "NAsm",
                   method.tau = "DL")
m1.plogit <- update(m1.pft, sm = "PLOGIT",backtransf = T)
# subgroup analysis by UN subregion
m1.plogits<- update(m1.pft, byvar= dat$UN.Sub.Region,print.byvar=F)
# plot forest plot
pdf("figs/Figure2.pdf", width = 12, height = 25)
forest(m1.plogits,
       leftlabs = c("Country, Year of survey", "Stunted", "Total"),
       digits = 2,
       col.by="black",
       #sortvar = Year,
       squaresize=0.5,
       col.square="navy",
       col.diamond="maroon",
       col.diamond.lines="maroon",
       print.pval.Q = T,
       xlab="Prevalence of stunting (%)",
       xlim = c(0, 60), at = c(0,20,40,60),pscale = 100,
       backtransf = T)
dev.off()
```


```
null device 
          1
```

# wasting meta-analysis: Figure 2


```
# Fit model
m2.pft <- metaprop(wasted_n, Weight_Height_N, data = dat, studlab = paste (Country, Year, sep = ", "),comb.fixed=F,
                   sm = "PFT", pscale = 100, method.ci = "NAsm",
                   method.tau = "DL")
m2.plogit <- update(m2.pft , sm = "PLOGIT",backtransf = T)
# subgroup analysis by UN subregion
m2.plogits<- update(m2.pft, byvar= dat$UN.Sub.Region,print.byvar=F)
# plot forest plot
pdf("figs/Figure3.pdf", width = 12, height = 25)
forest(m2.plogits,
       leftlabs = c("Country, Year of survey", "Wasted", "Total"),
       digits = 2,
       col.by="black",
       sortvar = Year,
       squaresize=0.5,
       col.square="navy",
       col.diamond="maroon",
       col.diamond.lines="maroon",
       print.pval.Q = T,
       xlab="Prevalence of wasting (%)",
       xlim = c(0, 25), at = c(0,5,10,15,20,25),pscale = 100,
       backtransf = T)
dev.off()
```


```
null device 
          1
```

# underweight meta-analysis: Figure 3


```
# Fit model
m3.pft <- metaprop(underweight_n, Weight_Age_N, data = dat, studlab = paste (Country, Year, sep = ", ") ,comb.fixed=F,
                   sm = "PFT", pscale = 100, method.ci = "NAsm",
                   method.tau = "DL")
m3.plogit <- update(m1.plogit , sm = "PLOGIT",backtransf = T)
# subgroup analysis by UN subregion
m3.plogits<- update(m3.pft, byvar= dat$UN.Sub.Region,print.byvar=F)
# plot forest plot
pdf("figs/Figure4.pdf", width = 12, height = 25)
forest(m3.plogits,
       leftlabs = c("Country, Year of survey", "Underweight", "Total"),
       digits = 2,
       col.by="black",
       sortvar = Year,
       squaresize=0.5,
       col.square="navy",
       col.diamond="maroon",
       col.diamond.lines="maroon",
       print.pval.Q = T,
       xlab="Prevalence of underweight (%)",
       xlim = c(0, 50), at = c(0,10,20,30,40,50),pscale = 100,
       backtransf = T)
dev.off()
```


```
null device 
          1
```

# stunting meta-analysis: Supplementary Figure S2


```
# Fit model
m1.pft <- metaprop(stunt_n, Height_Age_N, data = dat, studlab = paste (Country, Year, sep = ", "), comb.fixed =F,
                   sm = "PFT", pscale = 100, method.ci = "NAsm",
                   method.tau = "DL")
m1.plogit <- update(m1.pft, sm = "PLOGIT",backtransf = T)
# subgroup analysis by UN subregion
m1.plogits<- update(m1.pft, byvar= dat$UN.Regions,print.byvar=F)
# plot forest plot
pdf("figs/SupplementaryFigureS5.pdf", width = 12, height = 18)
forest(m1.plogits,
       leftlabs = c("Country, Year of survey", "Stunted", "Total"),
       digits = 2,
       col.by="black",
       #sortvar = Year,
       squaresize=0.5,
       col.square="navy",
       col.diamond="maroon",
       col.diamond.lines="maroon",
       print.pval.Q = T,
       xlab="Prevalence of stunting (%)",
       xlim = c(0, 60), at = c(0,20,40,60),pscale = 100,
       backtransf = T)
dev.off()
```


```
null device 
          1
```

# wasting meta-analysis: Supplementary Figure S3


```
# Fit model
m2.pft <- metaprop(wasted_n, Weight_Height_N, data = dat, studlab = paste (Country, Year, sep = ", "),comb.fixed=F,
                   sm = "PFT", pscale = 100, method.ci = "NAsm",
                   method.tau = "DL")
m2.plogit <- update(m2.pft , sm = "PLOGIT",backtransf = T)
# subgroup analysis by UN subregion
m2.plogits<- update(m2.plogit, byvar= UN.Regions,print.byvar=F)
# plot forest plot
pdf("figs/SupplementaryFigureS6.pdf", width = 12, height = 18)
forest(m2.plogits,
       leftlabs = c("Country, Year of survey", "Wasted", "Total"),
       digits = 2,
       col.by="black",
       sortvar = Year,
       squaresize=0.5,
       col.square="navy",
       col.diamond="maroon",
       col.diamond.lines="maroon",
       print.pval.Q = T,
       xlab="Prevalence of wasting (%)",
       xlim = c(0, 25), at = c(0,5,10,15,20,25),pscale = 100,
       backtransf = T)
dev.off()
```


```
null device 
          1
```

# Underweight meta-analysis: Supplementary Figure S4


```
# Fit model
m3.pft <- metaprop(underweight_n, Weight_Age_N, data = dat, studlab = paste (Country, Year, sep = ", ") ,comb.fixed=F,
                   sm = "PFT", pscale = 100, method.ci = "NAsm",
                   method.tau = "DL")
m3.plogit <- update(m3.pft, sm = "PLOGIT",backtransf = T)
# subgroup analysis by UN subregion
m3.plogits<- update(m3.plogit, byvar= UN.Regions,print.byvar=F)
# plot forest plot
pdf("figs/SupplementaryFigureS7.pdf", width = 15, height = 22)
forest(m3.plogits,
       leftlabs = c("Country, Year of survey", "Underweight", "Total"),
       digits = 2,
       col.by="black",
       sortvar = Year,
       squaresize=0.5,
       col.square="navy",
       col.diamond="maroon",
       col.diamond.lines="maroon",
       print.pval.Q = T,
       xlab="Prevalence of underweight (%)",
       xlim = c(0, 50), at = c(0,10,20,30,40,50),pscale = 100,
       backtransf = T)
dev.off()
```


```
null device 
          1
```


#Meta-regression. Fitting generalized linear mixed effects models for stunting


```
# Use glmm to estimate the risk of stunting
model_S <- rma.glmm(xi=stunt_n, ni=Height_Age_N, measure="PLO", mods = ~ relevel(factor(UN.Sub.Region),ref="Central Asia")+dat$HDI_Cat2, dat=dat, method="ML")
round(exp(coef(summary(model_S))[-1,c("estimate", "ci.lb", "ci.ub")]), 2)
```


```
print(model_S,digits=3)
```


```
Mixed-Effects Model (k = 62; tau^2 estimator: ML)

tau^2 (estimated amount of residual heterogeneity):     0.140
tau (square root of estimated tau^2 value):             0.374
I^2 (residual heterogeneity / unaccounted variability): 99.470%
H^2 (unaccounted variability / sampling variability):   188.763

Tests for Residual Heterogeneity:
Wld(df = 49) = 10531.472, p-val < .001
LRT(df = 49) = 11194.714, p-val < .001

Test of Moderators (coefficients 2:13):
QM(df = 12) = 82.263, p-val < .001

Model Results:

                                                                                     estimate     se    zval   pval   ci.lb   ci.ub 
intrcpt                                                                                -0.533  0.324  -1.645  0.100  -1.168   0.102    . 
relevel(factor(UN.Sub.Region), ref = "Central Asia")Eastern Africa                      0.632  0.295   2.142  0.032   0.054   1.211    * 
relevel(factor(UN.Sub.Region), ref = "Central Asia")Latin America and the Caribbean     0.346  0.299   1.157  0.247  -0.240   0.933      
relevel(factor(UN.Sub.Region), ref = "Central Asia")Middle Africa                       0.600  0.302   1.989  0.047   0.009   1.192    * 
relevel(factor(UN.Sub.Region), ref = "Central Asia")Northern Africa                     0.749  0.469   1.598  0.110  -0.170   1.667      
relevel(factor(UN.Sub.Region), ref = "Central Asia")Polynesia                           1.060  0.469   2.261  0.024   0.141   1.979    * 
relevel(factor(UN.Sub.Region), ref = "Central Asia")Southeastern Asia                   0.945  0.343   2.757  0.006   0.273   1.617   ** 
relevel(factor(UN.Sub.Region), ref = "Central Asia")Southern Africa                     0.608  0.326   1.864  0.062  -0.031   1.246    . 
relevel(factor(UN.Sub.Region), ref = "Central Asia")Southern Asia                       0.878  0.315   2.788  0.005   0.261   1.495   ** 
relevel(factor(UN.Sub.Region), ref = "Central Asia")Southern Europe                    -0.014  0.473  -0.030  0.976  -0.940   0.912      
relevel(factor(UN.Sub.Region), ref = "Central Asia")Western Africa                      0.308  0.295   1.044  0.296  -0.270   0.886      
relevel(factor(UN.Sub.Region), ref = "Central Asia")Western Asia                        0.366  0.329   1.111  0.266  -0.280   1.012      
dat$HDI_Cat2                                                                           -0.506  0.093  -5.462  <.001  -0.687  -0.324  *** 

---
Signif. codes:  0 ‘***’ 0.001 ‘**’ 0.01 ‘*’ 0.05 ‘.’ 0.1 ‘ ’ 1
```


```
# add malaria but subset to SSA
model_S <- rma.glmm(xi=stunt_n, ni=Height_Age_N, measure="PLO", mods = ~ relevel(factor(UN.Sub.Region),ref="Central Asia")+HDI_Cat2+scale(Mal_Prev), dat=dat,subset = SSA == "Sub-Saharan Africa", method="ML")
```


```
Redundant predictors dropped from the model.
```


```
round(exp(coef(summary(model_S))[-1,c("estimate", "ci.lb", "ci.ub")]), 2)
```


```
print(model_S,digits=3)
```


```
Mixed-Effects Model (k = 37; tau^2 estimator: ML)

tau^2 (estimated amount of residual heterogeneity):     0.088
tau (square root of estimated tau^2 value):             0.296
I^2 (residual heterogeneity / unaccounted variability): 99.199%
H^2 (unaccounted variability / sampling variability):   124.833

Tests for Residual Heterogeneity:
Wld(df = 31) = 4515.445, p-val < .001
LRT(df = 31) = 4609.480, p-val < .001

Test of Moderators (coefficients 2:6):
QM(df = 5) = 28.492, p-val < .001

Model Results:

                                                                     estimate     se    zval   pval   ci.lb   ci.ub 
intrcpt                                                                -0.601  0.170  -3.529  <.001  -0.936  -0.267  *** 
relevel(factor(UN.Sub.Region), ref = "Central Asia")Eastern Africa      0.431  0.129   3.346  <.001   0.178   0.683  *** 
relevel(factor(UN.Sub.Region), ref = "Central Asia")Middle Africa       0.234  0.157   1.498  0.134  -0.072   0.541      
relevel(factor(UN.Sub.Region), ref = "Central Asia")Southern Africa     0.383  0.209   1.834  0.067  -0.026   0.793    . 
HDI_Cat2                                                               -0.309  0.104  -2.956  0.003  -0.513  -0.104   ** 
scale(Mal_Prev)                                                         0.138  0.060   2.309  0.021   0.021   0.255    * 

---
Signif. codes:  0 ‘***’ 0.001 ‘**’ 0.01 ‘*’ 0.05 ‘.’ 0.1 ‘ ’ 1
```


Meta-regression. Fitting generalized linear mixed effects models for wasting


```
# Use glmm to estimate the risk of wasting
model_W <- rma.glmm( xi=wasted_n, ni=Weight_Height_N, measure="PLO", mods = ~ relevel(factor(UN.Sub.Region),ref="Central Asia")+dat$HDI_Cat2, dat=dat, method="ML")
```


```
Studies with NAs omitted from model fitting.Some yi/vi values are NA.Model failed to converge with max|grad| = 0.00128947 (tol = 0.001, component 1)
```


```
round(exp(coef(summary(model_W))[-1,c("estimate", "ci.lb", "ci.ub")]), 2)
```


```
print(model_W,digits=3)
```


```
Mixed-Effects Model (k = 61; tau^2 estimator: ML)

tau^2 (estimated amount of residual heterogeneity):     0.217
tau (square root of estimated tau^2 value):             0.466
I^2 (residual heterogeneity / unaccounted variability): 98.902%
H^2 (unaccounted variability / sampling variability):   91.058

Tests for Residual Heterogeneity:
Wld(df = 48) = 5254.887, p-val < .001
LRT(df = 48) = 5890.305, p-val < .001

Test of Moderators (coefficients 2:13):
QM(df = 12) = 148.361, p-val < .001

Model Results:

                                                                                     estimate     se    zval   pval   ci.lb   ci.ub 
intrcpt                                                                                -2.293  0.408  -5.622  <.001  -3.092  -1.493  *** 
relevel(factor(UN.Sub.Region), ref = "Central Asia")Eastern Africa                     -0.135  0.373  -0.361  0.718  -0.865   0.596      
relevel(factor(UN.Sub.Region), ref = "Central Asia")Latin America and the Caribbean    -0.788  0.377  -2.092  0.036  -1.527  -0.050    * 
relevel(factor(UN.Sub.Region), ref = "Central Asia")Middle Africa                       0.451  0.379   1.188  0.235  -0.293   1.195      
relevel(factor(UN.Sub.Region), ref = "Central Asia")Northern Africa                     1.281  0.586   2.186  0.029   0.132   2.430    * 
relevel(factor(UN.Sub.Region), ref = "Central Asia")Polynesia                          -0.586  0.593  -0.989  0.323  -1.748   0.575      
relevel(factor(UN.Sub.Region), ref = "Central Asia")Southeastern Asia                   1.189  0.430   2.765  0.006   0.346   2.032   ** 
relevel(factor(UN.Sub.Region), ref = "Central Asia")Southern Africa                    -0.199  0.413  -0.483  0.629  -1.008   0.610      
relevel(factor(UN.Sub.Region), ref = "Central Asia")Southern Asia                       1.251  0.395   3.164  0.002   0.476   2.025   ** 
relevel(factor(UN.Sub.Region), ref = "Central Asia")Southern Europe                    -0.540  0.611  -0.885  0.376  -1.737   0.657      
relevel(factor(UN.Sub.Region), ref = "Central Asia")Western Africa                      0.502  0.371   1.355  0.176  -0.224   1.229      
relevel(factor(UN.Sub.Region), ref = "Central Asia")Western Asia                        1.064  0.413   2.573  0.010   0.254   1.874    * 
dat$HDI_Cat2                                                                           -0.460  0.117  -3.931  <.001  -0.689  -0.230  *** 

---
Signif. codes:  0 ‘***’ 0.001 ‘**’ 0.01 ‘*’ 0.05 ‘.’ 0.1 ‘ ’ 1
```


```
# add malaria but subset to SSA
model_W <- rma.glmm(xi=wasted_n, ni=Weight_Height_N, measure="PLO", mods = ~ relevel(factor(UN.Sub.Region),ref="Central Asia")+HDI_Cat2+scale(Mal_Prev), dat=dat,subset = SSA == "Sub-Saharan Africa", method="ML")
```


```
Studies with NAs omitted from model fitting.Some yi/vi values are NA.Redundant predictors dropped from the model.
```


```
round(exp(coef(summary(model_W))[-1,c("estimate", "ci.lb", "ci.ub")]), 2)
```


```
print(model_W,digits=3)
```


```
Mixed-Effects Model (k = 36; tau^2 estimator: ML)

tau^2 (estimated amount of residual heterogeneity):     0.209
tau (square root of estimated tau^2 value):             0.457
I^2 (residual heterogeneity / unaccounted variability): 98.943%
H^2 (unaccounted variability / sampling variability):   94.609

Tests for Residual Heterogeneity:
Wld(df = 30) = 3499.916, p-val < .001
LRT(df = 30) = 3881.053, p-val < .001

Test of Moderators (coefficients 2:6):
QM(df = 5) = 30.649, p-val < .001

Model Results:

                                                                     estimate     se    zval   pval   ci.lb   ci.ub 
intrcpt                                                                -1.788  0.267  -6.696  <.001  -2.311  -1.265  *** 
relevel(factor(UN.Sub.Region), ref = "Central Asia")Eastern Africa     -0.669  0.202  -3.315  <.001  -1.065  -0.274  *** 
relevel(factor(UN.Sub.Region), ref = "Central Asia")Middle Africa      -0.091  0.243  -0.373  0.709  -0.566   0.385      
relevel(factor(UN.Sub.Region), ref = "Central Asia")Southern Africa    -0.785  0.327  -2.402  0.016  -1.426  -0.144    * 
HDI_Cat2                                                               -0.431  0.164  -2.625  0.009  -0.752  -0.109   ** 
scale(Mal_Prev)                                                        -0.032  0.093  -0.347  0.729  -0.214   0.150      

---
Signif. codes:  0 ‘***’ 0.001 ‘**’ 0.01 ‘*’ 0.05 ‘.’ 0.1 ‘ ’ 1
```


Meta-regression. Fitting generalized linear mixed effects models for underweight


```
# Use glmm to estimate the risk of underweight
model_U <- rma.glmm( xi=underweight_n, ni=Weight_Age_N, measure="PLO", mods = ~ relevel(factor(UN.Sub.Region),ref="Central Asia")+dat$HDI_Cat2, dat=dat, method="ML")
```


```
Studies with NAs omitted from model fitting.Some yi/vi values are NA.
```


```
round(exp(coef(summary(model_U))[-1,c("estimate", "ci.lb", "ci.ub")]), 2)
```


```
print(model_U,digits=3)
```


```
Mixed-Effects Model (k = 61; tau^2 estimator: ML)

tau^2 (estimated amount of residual heterogeneity):     0.163
tau (square root of estimated tau^2 value):             0.404
I^2 (residual heterogeneity / unaccounted variability): 99.275%
H^2 (unaccounted variability / sampling variability):   137.865

Tests for Residual Heterogeneity:
Wld(df = 48) = 6901.787, p-val < .001
LRT(df = 48) = 7289.439, p-val < .001

Test of Moderators (coefficients 2:13):
QM(df = 12) = 201.936, p-val < .001

Model Results:

                                                                                     estimate     se    zval   pval   ci.lb   ci.ub 
intrcpt                                                                                -1.502  0.353  -4.256  <.001  -2.194  -0.811  *** 
relevel(factor(UN.Sub.Region), ref = "Central Asia")Eastern Africa                      0.580  0.323   1.794  0.073  -0.053   1.213    . 
relevel(factor(UN.Sub.Region), ref = "Central Asia")Latin America and the Caribbean     0.448  0.326   1.374  0.170  -0.191   1.087      
relevel(factor(UN.Sub.Region), ref = "Central Asia")Middle Africa                       0.871  0.329   2.647  0.008   0.226   1.516   ** 
relevel(factor(UN.Sub.Region), ref = "Central Asia")Northern Africa                     0.794  0.509   1.561  0.119  -0.203   1.792      
relevel(factor(UN.Sub.Region), ref = "Central Asia")Polynesia                           0.535  0.510   1.048  0.295  -0.466   1.535      
relevel(factor(UN.Sub.Region), ref = "Central Asia")Southeastern Asia                   1.919  0.373   5.151  <.001   1.189   2.650  *** 
relevel(factor(UN.Sub.Region), ref = "Central Asia")Southern Africa                     0.501  0.356   1.406  0.160  -0.197   1.198      
relevel(factor(UN.Sub.Region), ref = "Central Asia")Southern Asia                       2.007  0.343   5.858  <.001   1.336   2.679  *** 
relevel(factor(UN.Sub.Region), ref = "Central Asia")Southern Europe                    -0.633  0.537  -1.177  0.239  -1.686   0.420      
relevel(factor(UN.Sub.Region), ref = "Central Asia")Western Africa                      0.852  0.321   2.652  0.008   0.223   1.482   ** 
relevel(factor(UN.Sub.Region), ref = "Central Asia")Western Asia                        0.928  0.360   2.579  0.010   0.223   1.633   ** 
dat$HDI_Cat2                                                                           -0.712  0.101  -7.068  <.001  -0.910  -0.515  *** 

---
Signif. codes:  0 ‘***’ 0.001 ‘**’ 0.01 ‘*’ 0.05 ‘.’ 0.1 ‘ ’ 1
```


```
# add malaria but subset to SSA
model_U <- rma.glmm(xi=underweight_n, ni=Weight_Age_N, measure="PLO", mods = ~ relevel(factor(UN.Sub.Region),ref="Central Asia")+HDI_Cat2+scale(Mal_Prev), dat=dat,subset = SSA == "Sub-Saharan Africa", method="ML")
```


```
Studies with NAs omitted from model fitting.Some yi/vi values are NA.Redundant predictors dropped from the model.
```


```
round(exp(coef(summary(model_U))[-1,c("estimate", "ci.lb", "ci.ub")]), 2)
```


```
print(model_U,digits=3)
```


```
Mixed-Effects Model (k = 36; tau^2 estimator: ML)

tau^2 (estimated amount of residual heterogeneity):     0.167
tau (square root of estimated tau^2 value):             0.408
I^2 (residual heterogeneity / unaccounted variability): 99.336%
H^2 (unaccounted variability / sampling variability):   150.671

Tests for Residual Heterogeneity:
Wld(df = 30) = 4511.691, p-val < .001
LRT(df = 30) = 4663.043, p-val < .001

Test of Moderators (coefficients 2:6):
QM(df = 5) = 34.427, p-val < .001

Model Results:

                                                                     estimate     se    zval   pval   ci.lb   ci.ub 
intrcpt                                                                -0.997  0.237  -4.206  <.001  -1.461  -0.532  *** 
relevel(factor(UN.Sub.Region), ref = "Central Asia")Eastern Africa     -0.228  0.180  -1.269  0.205  -0.580   0.124      
relevel(factor(UN.Sub.Region), ref = "Central Asia")Middle Africa      -0.090  0.216  -0.417  0.677  -0.514   0.334      
relevel(factor(UN.Sub.Region), ref = "Central Asia")Southern Africa    -0.389  0.289  -1.346  0.178  -0.954   0.177      
HDI_Cat2                                                               -0.489  0.145  -3.366  <.001  -0.773  -0.204  *** 
scale(Mal_Prev)                                                         0.083  0.083   1.004  0.315  -0.079   0.245      

---
Signif. codes:  0 ‘***’ 0.001 ‘**’ 0.01 ‘*’ 0.05 ‘.’ 0.1 ‘ ’ 1
```

# plot for HDI: linear trend + confidence interval


```
# plot stunting as function of HDI
s1=ggplot(dat, aes(x=HDI_2018, y=stunt)) +
  geom_point(alpha=0.8, size=3) +
  geom_smooth(method=lm , color="red", fill="#69b3a2", se=TRUE) +
  theme_ipsum()
s1=s1+ xlab("Human development index")+
  ylab('Prevalence of stunting (%)')+
  theme(
    axis.title.x=element_text(angle=0, color='black',face = "bold",size = 20),
    axis.title.y=element_text(angle=90, color='black', face='bold', size=20),
    axis.text.x = element_text(face="bold", color="black",size=20, angle=0),
    axis.text.y = element_text(face="bold", color="black", size=20, angle=0),
    legend.text = element_text(colour="black", size=20,face="bold"),
    legend.title = element_text(colour="black", size=20,face="bold"))
s1
```


```
cor.test(dat$HDI_2018, dat$stunt, method=c("spearman"))
```


```
Cannot compute exact p-value with ties
```


```
    Spearman's rank correlation rho

data:  dat$HDI_2018 and dat$stunt
S = 65609, p-value = 9.346e-09
alternative hypothesis: true rho is not equal to 0
sample estimates:
       rho 
-0.6521597
```


```
# plot wasting
w1=ggplot(dat, aes(x=HDI_2018, y=wasted)) +
  geom_point(alpha=0.8, size=3) +
  geom_smooth(method=lm , color="red", fill="#69b3a2", se=TRUE) +
  theme_ipsum()
w1=w1+ xlab("Human development index")+
  ylab('Prevalence of wasting (%)')+
  theme(
    axis.title.x=element_text(angle=0, color='black',face = "bold",size = 20),
    axis.title.y=element_text(angle=90, color='black', face='bold', size=20),
    axis.text.x = element_text(face="bold", color="black",size=20, angle=0),
    axis.text.y = element_text(face="bold", color="black", size=20, angle=0),
    legend.text = element_text(colour="black", size=20,face="bold"),
    legend.title = element_text(colour="black", size=20,face="bold"))
w1
```


```
cor.test(dat$HDI_2018, dat$wasted, method=c("spearman"))
```


```
Cannot compute exact p-value with ties
```


```
    Spearman's rank correlation rho

data:  dat$HDI_2018 and dat$wasted
S = 54067, p-value = 0.0005505
alternative hypothesis: true rho is not equal to 0
sample estimates:
       rho 
-0.4295821
```


```
# plot underweight
u1=ggplot(dat, aes(x=HDI_2018, y=underweight)) +
  geom_point(alpha=0.8, size=3) +
  geom_smooth(method=lm , color="red", fill="#69b3a2", se=TRUE) +
  theme_ipsum()
u1=u1+ xlab("Human development index")+
  ylab('Prevalence of underweight (%)')+
  theme(
    axis.title.x=element_text(angle=0, color='black',face = "bold",size = 20),
    axis.title.y=element_text(angle=90, color='black', face='bold', size=20),
    axis.text.x = element_text(face="bold", color="black",size=20, angle=0),
    axis.text.y = element_text(face="bold", color="black", size=20, angle=0),
    legend.text = element_text(colour="black", size=20,face="bold"),
    legend.title = element_text(colour="black", size=20,face="bold"))
u1
```


```
cor.test(dat$HDI_2018, dat$underweight, method=c("spearman"))
```


```
Cannot compute exact p-value with ties
```


```
    Spearman's rank correlation rho

data:  dat$HDI_2018 and dat$underweight
S = 63249, p-value = 2.959e-09
alternative hypothesis: true rho is not equal to 0
sample estimates:
       rho 
-0.6723614
```

# plot for Malaria: linear trend + confidence interval


```
# plot stunting as function of malaria
s1=ggplot(subset(dat, UN.Regions %in% c("Africa")), aes(x=Mal_Prev, y=stunt)) +
  geom_point(alpha=0.8, size=3) +
  geom_smooth(method=lm , color="red", fill="#69b3a2", se=TRUE) +
  theme_ipsum()
s1=s1+ xlab("Malaria prevalence (%)")+
  ylab('Prevalence of stunting (%)')+
  theme(
    axis.title.x=element_text(angle=0, color='black',face = "bold",size = 20),
    axis.title.y=element_text(angle=90, color='black', face='bold', size=20),
    axis.text.x = element_text(face="bold", color="black",size=20, angle=0),
    axis.text.y = element_text(face="bold", color="black", size=20, angle=0),
    legend.text = element_text(colour="black", size=20,face="bold"),
    legend.title = element_text(colour="black", size=20,face="bold"))
s1
```


```
cor.test(dat$Mal2, dat$stunt, method=c("spearman"))
```


```
Cannot compute exact p-value with ties
```


```
    Spearman's rank correlation rho

data:  dat$Mal2 and dat$stunt
S = 5269.7, p-value = 0.02207
alternative hypothesis: true rho is not equal to 0
sample estimates:
      rho 
0.3753335
```


```
# plot wasting
w1=ggplot(subset(dat, UN.Regions %in% c("Africa")), aes(x=Mal_Prev, y=wasted)) +
  geom_point(alpha=0.8, size=3) +
  geom_smooth(method=lm , color="red", fill="#69b3a2", se=TRUE) +
  theme_ipsum()
w1=w1+ xlab("Malaria prevalence (%)")+
  ylab('Prevalence of wasting (%)')+
  theme(
    axis.title.x=element_text(angle=0, color='black',face = "bold",size = 20),
    axis.title.y=element_text(angle=90, color='black', face='bold', size=20),
    axis.text.x = element_text(face="bold", color="black",size=20, angle=0),
    axis.text.y = element_text(face="bold", color="black", size=20, angle=0),
    legend.text = element_text(colour="black", size=20,face="bold"),
    legend.title = element_text(colour="black", size=20,face="bold"))
w1
```


```
cor.test(dat$Mal2, dat$wasted, method=c("spearman"))
```


```
Cannot compute exact p-value with ties
```


```
    Spearman's rank correlation rho

data:  dat$Mal2 and dat$wasted
S = 4857.5, p-value = 0.02429
alternative hypothesis: true rho is not equal to 0
sample estimates:
     rho 
0.374839
```


```
# plot underweight
u1=ggplot(subset(dat, UN.Regions %in% c("Africa")), aes(x=Mal_Prev, y=underweight)) +
  geom_point(alpha=0.8, size=3) +
  geom_smooth(method=lm , color="red", fill="#69b3a2", se=TRUE) +
  theme_ipsum()
u1=u1+ xlab("Malaria prevalence (%)")+
  ylab('Prevalence of underweight (%)')+
  theme(
    axis.title.x=element_text(angle=0, color='black',face = "bold",size = 20),
    axis.title.y=element_text(angle=90, color='black', face='bold', size=20),
    axis.text.x = element_text(face="bold", color="black",size=20, angle=0),
    axis.text.y = element_text(face="bold", color="black", size=20, angle=0),
    legend.text = element_text(colour="black", size=20,face="bold"),
    legend.title = element_text(colour="black", size=20,face="bold"))
u1
```


```
cor.test(dat$Mal2, dat$underweight, method=c("spearman"))
```


```
Cannot compute exact p-value with ties
```


```
    Spearman's rank correlation rho

data:  dat$Mal2 and dat$underweight
S = 3302, p-value = 0.000244
alternative hypothesis: true rho is not equal to 0
sample estimates:
      rho 
0.5750338
```


```
# Use row names as point labels
#df$name <- rownames(df)
ed=ggscatter(dat, x = "HDI_2018", y = "Value", palette = "jco", add = "reg.line", conf.int = TRUE,
   label = "Country", repel = TRUE)+
  stat_cor(aes(), method = "spearman", label.y = 90)+
  theme_ipsum()
ed=ed+ xlab("Human Development Index")+
  ylab('Education Attainment for Women')+
  theme(
    axis.title.x=element_text(angle=0, color='black',face = "bold",size = 14),
    axis.title.y=element_text(angle=90, color='black', face='bold', size=14),
    axis.text.x = element_text(face="bold", color="black",size=20, angle=0),
    axis.text.y = element_text(face="bold", color="black", size=20, angle=0),
    legend.text = element_text(colour="black", size=20,face="bold"),
    legend.title = element_text(colour="black", size=20,face="bold"))
ed
```

LS0tCnRpdGxlOiBSIFNjcmlwdCB0byByZXJvZHVjZSByZXN1bHRzIG9mIDtHbG9iYWwsIHJlZ2lvbmFsIGFuZCBjb3VudHJ5IGVwaWRlbWlvbG9neQogIGFuZCBwcmV2YWxlbmNlIG9mIHN0dW50aW5nLCB3YXN0aW5nIGFuZCB1bmRlcndlaWdodCBpbiBsb3ctIGFuZCBtaWRkbGUtIGluY29tZSBjb3VudHJpZXMsCiAgMjAwNi0yMDE4Cm91dHB1dDoKICBodG1sX2RvY3VtZW50OgogICAgZGZfcHJpbnQ6IHBhZ2VkCiAgaHRtbF9ub3RlYm9vazogZGVmYXVsdAogIHdvcmRfZG9jdW1lbnQ6IGRlZmF1bHQKLS0tCgojIGNsZWFyIFIgZ2xvYmFsIGVudmlyb25tZW50CnJtKGxpc3Q9bHMoKSkKCiNMb2FkIHRoZSBsaWJyYXJpZXMKCmBgYHtyLCBtZXNzYWdlPUZBTFNFfQojIGxvYWQgbGlicmFyaWVzCmxpYnJhcnkob2xzcnIpICMgbGluZWFyIHJlZ3Jlc3Npb24KbGlicmFyeSh0aWR5dmVyc2UpICMgZ2VuZXJhbCBzdHVmZgpsaWJyYXJ5KGhyYnJ0aGVtZXMpICMgcHJldHR5IHBsb3RzCmxpYnJhcnkoZ2dwbG90MikgIyBwcmV0dHkgcGxvdHMKbGlicmFyeShwbG90bHkpICMgaW50ZXJhY3RpdmUgcGxvdHMKbGlicmFyeShnYXBtaW5kZXIpICMgaW50ZXJhY3RpdmUgcGxvdHMKbGlicmFyeShwYXN0ZWNzKSAjIHN0YXQuZGVzYwpsaWJyYXJ5KG1ldGFmb3IpICMgZm9yIG1ldGEtYW5hbHlzaXM6IG1peGVkLWVmZmVjdHMgbG9naXN0aWMgYW5kIFBvaXNzb24gcmVncmVzc2lvbiBtb2RlbHMKbGlicmFyeShtZXRhKSAjIG1ldGEtcmVncmVzc2lvbiwgR0xNTSwgZm9yZXN0IHBsb3RzLChzdWJncm91cCkgbWV0YS1hbmFseXNlcy4KbGlicmFyeShzcCkgIyBzcGF0aWFsIGRhdGEKbGlicmFyeShyZ2RhbCkgIyAgcHJvamVjdGlvbi90cmFuc2Zvcm1hdGlvbiBvcGVyYXRpb25zIGZvciBzaGFwZWZpbGVzCmxpYnJhcnkoc2YpICMgU2ltcGxlIEZlYXR1cmVzIGZvciBSCmxpYnJhcnkocm5hdHVyYWxlYXJ0aCkKbGlicmFyeSh0bWFwKSAjIGNvb2wgbWFwcwpsaWJyYXJ5KGdncHVicikgIyAnZ2dwbG90MicgQmFzZWQgUHVibGljYXRpb24gUmVhZHkgUGxvdHMKbGlicmFyeShnZ3BtaXNjKSAjTWlzY2VsbGFuZW91cyBFeHRlbnNpb25zIHRvICdnZ3Bsb3QyJwpsaWJyYXJ5KHNwRGF0YSkKbGlicmFyeShjb3dwbG90KSMgcGxvdCBncmlkCmBgYAoKIyBsb2FkIGRhdGEKCndvcmxkCmBgYHtyfQpkYXQgPC0gcmVhZC5jc3YoZmlsZT0iREhTNzdfTUVUQS5jc3YiLCBoZWFkZXI9VCxzdHJpbmdzQXNGYWN0b3JzPUYpCmhkaTwtIHJlYWQuY3N2KGZpbGU9IkhESS5jc3YiLCBoZWFkZXI9VCxzdHJpbmdzQXNGYWN0b3JzPUYpCmVkdWM8LXJlYWQuY3N2KGZpbGU9IldvbWVuX0VkdWNhdGlvbi5jc3YiKQpkYXQgPC0gbWVyZ2UoZGF0LGhkaSwgYnk9YygiQ291bnRyeSIpLCBhbGwueD1UKQpkYXQgPC0gbWVyZ2UoZGF0LGVkdWMsIGJ5PWMoIkNvdW50cnkiKSwgYWxsLng9VCkKCiNkYXRfbWV0YSA8LSBtZXJnZShkYXQsaGRpLCBieT1jKCJDb3VudHJ5IiksIGFsbC54PVQpCmRhdCRuYW1lPWRhdCRDb3VudHJ5CgpgYGAKCgoKIyBkaXNwbGF5IG1hcHMKCmBgYHtyIG1hcCBzdHVudH0KV29ybGREYXRhIDwtIG1hcF9kYXRhKCd3b3JsZCcpCldvcmxkRGF0YSAlPiUgZmlsdGVyKHJlZ2lvbiAhPSAiQW50YXJjdGljYSIpIC0+IFdvcmxkRGF0YQpXb3JsZERhdGEgPC0gZm9ydGlmeShXb3JsZERhdGEpCgpwIDwtIGdncGxvdCgpCnAgPC0gcCArIGdlb21fbWFwKGRhdGE9V29ybGREYXRhLCBtYXA9V29ybGREYXRhLAogICAgICAgICAgICAgICAgICBhZXMoeD1sb25nLCB5PWxhdCwgZ3JvdXA9Z3JvdXAsIG1hcF9pZD1yZWdpb24pLAogICAgICAgICAgICAgICAgICBmaWxsPSJ3aGl0ZSIsIGNvbG91cj0iIzdmN2Y3ZiIsIHNpemU9MC41KQpwIDwtIHAgKyBnZW9tX21hcChkYXRhPWRhdCwgbWFwPVdvcmxkRGF0YSwKICAgICAgICAgICAgICAgICAgYWVzKGZpbGw9c3R1bnQsIG1hcF9pZD1Db3VudHJ5KSwKICAgICAgICAgICAgICAgICAgY29sb3VyPSIjN2Y3ZjdmIiwgc2l6ZT0wLjUpCnAgPC0gcCArIGNvb3JkX21hcCgicmVjdGFuZ3VsYXIiLCBsYXQwPTAsIHhsaW09YygtMTgwLDE4MCksIHlsaW09YygtNjAsIDkwKSkKI3AgPC0gcCArIHNjYWxlX2ZpbGxfY29udGludW91cyhsb3c9InRoaXN0bGUyIiwgaGlnaD0iZGFya3JlZCIsIGd1aWRlPSJjb2xvcmJhciIpCnAgPC1wKyBzY2FsZV9maWxsX2Rpc3RpbGxlcihwYWxldHRlID0gIlNwZWN0cmFsIikKCnAgPC0gcCArIHNjYWxlX3lfY29udGludW91cyhicmVha3M9YygpKQpwIDwtIHAgKyBzY2FsZV94X2NvbnRpbnVvdXMoYnJlYWtzPWMoKSkKcCA8LSBwICsgbGFicyhmaWxsPSJTdHVudGluZyglKSIsIHRpdGxlPSIiLCB4PSIiLCB5PSIiKQpwIDwtIHAgKyB0aGVtZV9idygpCnAgPC0gcCArIHRoZW1lKHBhbmVsLmJvcmRlciA9IGVsZW1lbnRfYmxhbmsoKSkKCnBhcihtYWk9YygwLDAsMC4yLDApLHhheHM9ImkiLHlheHM9ImkiKQpwIAoKYGBgCgoKCgpgYGB7cn0Kd29ybGRfc3R1bnQgPSB3b3JsZCAlPiUKICBmaWx0ZXIoY29udGluZW50IT0gIkFudGFyY3RpY2EiLCAhaXMubmEoaXNvX2EyKSkgJT4lCiAgI2ZpbHRlcihjb250aW5lbnQgPT0gIkFmcmljYSIsICFpcy5uYShpc29fYTIpKSAjJT4lCiAgbGVmdF9qb2luKHdvcmxkYmFua19kZiwgYnkgPSAiaXNvX2EyIikgJT4lCiAgZHBseXI6OnNlbGVjdChuYW1lLCBzdWJyZWdpb24sIGdkcFBlcmNhcCwgSERJLCBwb3BfZ3Jvd3RoKSAlPiUKICBsZWZ0X2pvaW4oZGF0LCBieSA9ICJuYW1lIikgJT4lCiAgc3RfdHJhbnNmb3JtKCIrcHJvaj1sb25nbGF0ICtkYXR1bT1XR1M4NCArbm9fZGVmcyIpCgp3b3JsZGJhbmtfZGYKIyBNYXAgdGhlIG91dHB1dAp0bV9zaGFwZSh3b3JsZF9zdHVudCkgKyB0bV9wb2x5Z29ucyhjb2w9IkhESV8yMDE4Iiwgc3R5bGU9InF1YW50aWxlIikgKwogIHRtX2xlZ2VuZChvdXRzaWRlPVRSVUUpCmBgYAoKCiMgc3R1bnRpbmcgbWV0YS1hbmFseXNpczogRmlndXJlIDEKCmBgYHtyIHN0dW50aW5nfQojIEZpdCBtb2RlbAptMS5wZnQgPC0gbWV0YXByb3Aoc3R1bnRfbiwgSGVpZ2h0X0FnZV9OLCBkYXRhID0gZGF0LCBzdHVkbGFiID0gcGFzdGUgKENvdW50cnksIFllYXIsIHNlcCA9ICIsICIpLCBjb21iLmZpeGVkID1GLAogICAgICAgICAgICAgICAgICAgc20gPSAiUEZUIiwgcHNjYWxlID0gMTAwLCBtZXRob2QuY2kgPSAiTkFzbSIsCiAgICAgICAgICAgICAgICAgICBtZXRob2QudGF1ID0gIkRMIikKbTEucGxvZ2l0IDwtIHVwZGF0ZShtMS5wZnQsIHNtID0gIlBMT0dJVCIsYmFja3RyYW5zZiA9IFQpCiMgc3ViZ3JvdXAgYW5hbHlzaXMgYnkgVU4gc3VicmVnaW9uCm0xLnBsb2dpdHM8LSB1cGRhdGUobTEucGZ0LCBieXZhcj0gZGF0JFVOLlN1Yi5SZWdpb24scHJpbnQuYnl2YXI9RikKCiMgcGxvdCBmb3Jlc3QgcGxvdApwZGYoImZpZ3MvRmlndXJlMi5wZGYiLCB3aWR0aCA9IDEyLCBoZWlnaHQgPSAyNSkKZm9yZXN0KG0xLnBsb2dpdHMsCiAgICAgICBsZWZ0bGFicyA9IGMoIkNvdW50cnksIFllYXIgb2Ygc3VydmV5IiwgIlN0dW50ZWQiLCAiVG90YWwiKSwKICAgICAgIGRpZ2l0cyA9IDIsCiAgICAgICBjb2wuYnk9ImJsYWNrIiwKICAgICAgICNzb3J0dmFyID0gWWVhciwKICAgICAgIHNxdWFyZXNpemU9MC41LAogICAgICAgY29sLnNxdWFyZT0ibmF2eSIsCiAgICAgICBjb2wuZGlhbW9uZD0ibWFyb29uIiwKICAgICAgIGNvbC5kaWFtb25kLmxpbmVzPSJtYXJvb24iLAogICAgICAgcHJpbnQucHZhbC5RID0gVCwKICAgICAgIHhsYWI9IlByZXZhbGVuY2Ugb2Ygc3R1bnRpbmcgKCUpIiwKICAgICAgIHhsaW0gPSBjKDAsIDYwKSwgYXQgPSBjKDAsMjAsNDAsNjApLHBzY2FsZSA9IDEwMCwKICAgICAgIGJhY2t0cmFuc2YgPSBUKQpkZXYub2ZmKCkKCmBgYAoKCiMgd2FzdGluZyBtZXRhLWFuYWx5c2lzOiBGaWd1cmUgMgoKYGBge3Igd2FzdGluZ30KIyBGaXQgbW9kZWwKCm0yLnBmdCA8LSBtZXRhcHJvcCh3YXN0ZWRfbiwgV2VpZ2h0X0hlaWdodF9OLCBkYXRhID0gZGF0LCBzdHVkbGFiID0gcGFzdGUgKENvdW50cnksIFllYXIsIHNlcCA9ICIsICIpLGNvbWIuZml4ZWQ9RiwKICAgICAgICAgICAgICAgICAgIHNtID0gIlBGVCIsIHBzY2FsZSA9IDEwMCwgbWV0aG9kLmNpID0gIk5Bc20iLAogICAgICAgICAgICAgICAgICAgbWV0aG9kLnRhdSA9ICJETCIpCm0yLnBsb2dpdCA8LSB1cGRhdGUobTIucGZ0ICwgc20gPSAiUExPR0lUIixiYWNrdHJhbnNmID0gVCkKIyBzdWJncm91cCBhbmFseXNpcyBieSBVTiBzdWJyZWdpb24KbTIucGxvZ2l0czwtIHVwZGF0ZShtMi5wZnQsIGJ5dmFyPSBkYXQkVU4uU3ViLlJlZ2lvbixwcmludC5ieXZhcj1GKQoKIyBwbG90IGZvcmVzdCBwbG90CnBkZigiZmlncy9GaWd1cmUzLnBkZiIsIHdpZHRoID0gMTIsIGhlaWdodCA9IDI1KQpmb3Jlc3QobTIucGxvZ2l0cywKICAgICAgIGxlZnRsYWJzID0gYygiQ291bnRyeSwgWWVhciBvZiBzdXJ2ZXkiLCAiV2FzdGVkIiwgIlRvdGFsIiksCiAgICAgICBkaWdpdHMgPSAyLAogICAgICAgY29sLmJ5PSJibGFjayIsCiAgICAgICBzb3J0dmFyID0gWWVhciwKICAgICAgIHNxdWFyZXNpemU9MC41LAogICAgICAgY29sLnNxdWFyZT0ibmF2eSIsCiAgICAgICBjb2wuZGlhbW9uZD0ibWFyb29uIiwKICAgICAgIGNvbC5kaWFtb25kLmxpbmVzPSJtYXJvb24iLAogICAgICAgcHJpbnQucHZhbC5RID0gVCwKICAgICAgIHhsYWI9IlByZXZhbGVuY2Ugb2Ygd2FzdGluZyAoJSkiLAogICAgICAgeGxpbSA9IGMoMCwgMjUpLCBhdCA9IGMoMCw1LDEwLDE1LDIwLDI1KSxwc2NhbGUgPSAxMDAsCiAgICAgICBiYWNrdHJhbnNmID0gVCkKZGV2Lm9mZigpCgpgYGAKCgojIHVuZGVyd2VpZ2h0IG1ldGEtYW5hbHlzaXM6IEZpZ3VyZSAzCgpgYGB7ciB1bmRlcndlaWdodH0KIyBGaXQgbW9kZWwKCm0zLnBmdCA8LSBtZXRhcHJvcCh1bmRlcndlaWdodF9uLCBXZWlnaHRfQWdlX04sIGRhdGEgPSBkYXQsIHN0dWRsYWIgPSBwYXN0ZSAoQ291bnRyeSwgWWVhciwgc2VwID0gIiwgIikgLGNvbWIuZml4ZWQ9RiwKICAgICAgICAgICAgICAgICAgIHNtID0gIlBGVCIsIHBzY2FsZSA9IDEwMCwgbWV0aG9kLmNpID0gIk5Bc20iLAogICAgICAgICAgICAgICAgICAgbWV0aG9kLnRhdSA9ICJETCIpCgptMy5wbG9naXQgPC0gdXBkYXRlKG0xLnBsb2dpdCAsIHNtID0gIlBMT0dJVCIsYmFja3RyYW5zZiA9IFQpCiMgc3ViZ3JvdXAgYW5hbHlzaXMgYnkgVU4gc3VicmVnaW9uCm0zLnBsb2dpdHM8LSB1cGRhdGUobTMucGZ0LCBieXZhcj0gZGF0JFVOLlN1Yi5SZWdpb24scHJpbnQuYnl2YXI9RikKCiMgcGxvdCBmb3Jlc3QgcGxvdApwZGYoImZpZ3MvRmlndXJlNC5wZGYiLCB3aWR0aCA9IDEyLCBoZWlnaHQgPSAyNSkKZm9yZXN0KG0zLnBsb2dpdHMsCiAgICAgICBsZWZ0bGFicyA9IGMoIkNvdW50cnksIFllYXIgb2Ygc3VydmV5IiwgIlVuZGVyd2VpZ2h0IiwgIlRvdGFsIiksCiAgICAgICBkaWdpdHMgPSAyLAogICAgICAgY29sLmJ5PSJibGFjayIsCiAgICAgICBzb3J0dmFyID0gWWVhciwKICAgICAgIHNxdWFyZXNpemU9MC41LAogICAgICAgY29sLnNxdWFyZT0ibmF2eSIsCiAgICAgICBjb2wuZGlhbW9uZD0ibWFyb29uIiwKICAgICAgIGNvbC5kaWFtb25kLmxpbmVzPSJtYXJvb24iLAogICAgICAgcHJpbnQucHZhbC5RID0gVCwKICAgICAgIHhsYWI9IlByZXZhbGVuY2Ugb2YgdW5kZXJ3ZWlnaHQgKCUpIiwKICAgICAgIHhsaW0gPSBjKDAsIDUwKSwgYXQgPSBjKDAsMTAsMjAsMzAsNDAsNTApLHBzY2FsZSA9IDEwMCwKICAgICAgIGJhY2t0cmFuc2YgPSBUKQpkZXYub2ZmKCkKCmBgYAoKCiMgc3R1bnRpbmcgbWV0YS1hbmFseXNpczogU3VwcGxlbWVudGFyeSBGaWd1cmUgUzIKCmBgYHtyIHN0dW50aW5nIFMyfQojIEZpdCBtb2RlbAptMS5wZnQgPC0gbWV0YXByb3Aoc3R1bnRfbiwgSGVpZ2h0X0FnZV9OLCBkYXRhID0gZGF0LCBzdHVkbGFiID0gcGFzdGUgKENvdW50cnksIFllYXIsIHNlcCA9ICIsICIpLCBjb21iLmZpeGVkID1GLAogICAgICAgICAgICAgICAgICAgc20gPSAiUEZUIiwgcHNjYWxlID0gMTAwLCBtZXRob2QuY2kgPSAiTkFzbSIsCiAgICAgICAgICAgICAgICAgICBtZXRob2QudGF1ID0gIkRMIikKbTEucGxvZ2l0IDwtIHVwZGF0ZShtMS5wZnQsIHNtID0gIlBMT0dJVCIsYmFja3RyYW5zZiA9IFQpCiMgc3ViZ3JvdXAgYW5hbHlzaXMgYnkgVU4gc3VicmVnaW9uCm0xLnBsb2dpdHM8LSB1cGRhdGUobTEucGZ0LCBieXZhcj0gZGF0JFVOLlJlZ2lvbnMscHJpbnQuYnl2YXI9RikKCiMgcGxvdCBmb3Jlc3QgcGxvdApwZGYoImZpZ3MvU3VwcGxlbWVudGFyeUZpZ3VyZVM1LnBkZiIsIHdpZHRoID0gMTIsIGhlaWdodCA9IDE4KQpmb3Jlc3QobTEucGxvZ2l0cywKICAgICAgIGxlZnRsYWJzID0gYygiQ291bnRyeSwgWWVhciBvZiBzdXJ2ZXkiLCAiU3R1bnRlZCIsICJUb3RhbCIpLAogICAgICAgZGlnaXRzID0gMiwKICAgICAgIGNvbC5ieT0iYmxhY2siLAogICAgICAgI3NvcnR2YXIgPSBZZWFyLAogICAgICAgc3F1YXJlc2l6ZT0wLjUsCiAgICAgICBjb2wuc3F1YXJlPSJuYXZ5IiwKICAgICAgIGNvbC5kaWFtb25kPSJtYXJvb24iLAogICAgICAgY29sLmRpYW1vbmQubGluZXM9Im1hcm9vbiIsCiAgICAgICBwcmludC5wdmFsLlEgPSBULAogICAgICAgeGxhYj0iUHJldmFsZW5jZSBvZiBzdHVudGluZyAoJSkiLAogICAgICAgeGxpbSA9IGMoMCwgNjApLCBhdCA9IGMoMCwyMCw0MCw2MCkscHNjYWxlID0gMTAwLAogICAgICAgYmFja3RyYW5zZiA9IFQpCmRldi5vZmYoKQoKYGBgCgojIHdhc3RpbmcgbWV0YS1hbmFseXNpczogU3VwcGxlbWVudGFyeSBGaWd1cmUgUzMKCmBgYHtyIHdhc3RpbmcgUzN9CiMgRml0IG1vZGVsCgptMi5wZnQgPC0gbWV0YXByb3Aod2FzdGVkX24sIFdlaWdodF9IZWlnaHRfTiwgZGF0YSA9IGRhdCwgc3R1ZGxhYiA9IHBhc3RlIChDb3VudHJ5LCBZZWFyLCBzZXAgPSAiLCAiKSxjb21iLmZpeGVkPUYsCiAgICAgICAgICAgICAgICAgICBzbSA9ICJQRlQiLCBwc2NhbGUgPSAxMDAsIG1ldGhvZC5jaSA9ICJOQXNtIiwKICAgICAgICAgICAgICAgICAgIG1ldGhvZC50YXUgPSAiREwiKQptMi5wbG9naXQgPC0gdXBkYXRlKG0yLnBmdCAsIHNtID0gIlBMT0dJVCIsYmFja3RyYW5zZiA9IFQpCiMgc3ViZ3JvdXAgYW5hbHlzaXMgYnkgVU4gc3VicmVnaW9uCm0yLnBsb2dpdHM8LSB1cGRhdGUobTIucGxvZ2l0LCBieXZhcj0gVU4uUmVnaW9ucyxwcmludC5ieXZhcj1GKQoKIyBwbG90IGZvcmVzdCBwbG90CnBkZigiZmlncy9TdXBwbGVtZW50YXJ5RmlndXJlUzYucGRmIiwgd2lkdGggPSAxMiwgaGVpZ2h0ID0gMTgpCmZvcmVzdChtMi5wbG9naXRzLAogICAgICAgbGVmdGxhYnMgPSBjKCJDb3VudHJ5LCBZZWFyIG9mIHN1cnZleSIsICJXYXN0ZWQiLCAiVG90YWwiKSwKICAgICAgIGRpZ2l0cyA9IDIsCiAgICAgICBjb2wuYnk9ImJsYWNrIiwKICAgICAgIHNvcnR2YXIgPSBZZWFyLAogICAgICAgc3F1YXJlc2l6ZT0wLjUsCiAgICAgICBjb2wuc3F1YXJlPSJuYXZ5IiwKICAgICAgIGNvbC5kaWFtb25kPSJtYXJvb24iLAogICAgICAgY29sLmRpYW1vbmQubGluZXM9Im1hcm9vbiIsCiAgICAgICBwcmludC5wdmFsLlEgPSBULAogICAgICAgeGxhYj0iUHJldmFsZW5jZSBvZiB3YXN0aW5nICglKSIsCiAgICAgICB4bGltID0gYygwLCAyNSksIGF0ID0gYygwLDUsMTAsMTUsMjAsMjUpLHBzY2FsZSA9IDEwMCwKICAgICAgIGJhY2t0cmFuc2YgPSBUKQpkZXYub2ZmKCkKCmBgYAoKCgojIFVuZGVyd2VpZ2h0IG1ldGEtYW5hbHlzaXM6IFN1cHBsZW1lbnRhcnkgRmlndXJlIFM0CgpgYGB7ciB1bmRlcndlaWdodCBTNH0KIyBGaXQgbW9kZWwKCm0zLnBmdCA8LSBtZXRhcHJvcCh1bmRlcndlaWdodF9uLCBXZWlnaHRfQWdlX04sIGRhdGEgPSBkYXQsIHN0dWRsYWIgPSBwYXN0ZSAoQ291bnRyeSwgWWVhciwgc2VwID0gIiwgIikgLGNvbWIuZml4ZWQ9RiwKICAgICAgICAgICAgICAgICAgIHNtID0gIlBGVCIsIHBzY2FsZSA9IDEwMCwgbWV0aG9kLmNpID0gIk5Bc20iLAogICAgICAgICAgICAgICAgICAgbWV0aG9kLnRhdSA9ICJETCIpCgptMy5wbG9naXQgPC0gdXBkYXRlKG0zLnBmdCwgc20gPSAiUExPR0lUIixiYWNrdHJhbnNmID0gVCkKIyBzdWJncm91cCBhbmFseXNpcyBieSBVTiBzdWJyZWdpb24KbTMucGxvZ2l0czwtIHVwZGF0ZShtMy5wbG9naXQsIGJ5dmFyPSBVTi5SZWdpb25zLHByaW50LmJ5dmFyPUYpCgojIHBsb3QgZm9yZXN0IHBsb3QKcGRmKCJmaWdzL1N1cHBsZW1lbnRhcnlGaWd1cmVTNy5wZGYiLCB3aWR0aCA9IDE1LCBoZWlnaHQgPSAyMikKZm9yZXN0KG0zLnBsb2dpdHMsCiAgICAgICBsZWZ0bGFicyA9IGMoIkNvdW50cnksIFllYXIgb2Ygc3VydmV5IiwgIlVuZGVyd2VpZ2h0IiwgIlRvdGFsIiksCiAgICAgICBkaWdpdHMgPSAyLAogICAgICAgY29sLmJ5PSJibGFjayIsCiAgICAgICBzb3J0dmFyID0gWWVhciwKICAgICAgIHNxdWFyZXNpemU9MC41LAogICAgICAgY29sLnNxdWFyZT0ibmF2eSIsCiAgICAgICBjb2wuZGlhbW9uZD0ibWFyb29uIiwKICAgICAgIGNvbC5kaWFtb25kLmxpbmVzPSJtYXJvb24iLAogICAgICAgcHJpbnQucHZhbC5RID0gVCwKICAgICAgIHhsYWI9IlByZXZhbGVuY2Ugb2YgdW5kZXJ3ZWlnaHQgKCUpIiwKICAgICAgIHhsaW0gPSBjKDAsIDUwKSwgYXQgPSBjKDAsMTAsMjAsMzAsNDAsNTApLHBzY2FsZSA9IDEwMCwKICAgICAgIGJhY2t0cmFuc2YgPSBUKQpkZXYub2ZmKCkKCmBgYAoKCgojTWV0YS1yZWdyZXNzaW9uLiBGaXR0aW5nIGdlbmVyYWxpemVkIGxpbmVhciBtaXhlZCBlZmZlY3RzIG1vZGVscyBmb3Igc3R1bnRpbmcKCmBgYHtyIHN0dW50aW5nIG1ldGEtcmVncmVzc2lvbn0KIyBVc2UgZ2xtbSB0byBlc3RpbWF0ZSB0aGUgcmlzayBvZiBzdHVudGluZwptb2RlbF9TIDwtIHJtYS5nbG1tKHhpPXN0dW50X24sIG5pPUhlaWdodF9BZ2VfTiwgbWVhc3VyZT0iUExPIiwgbW9kcyA9IH4gcmVsZXZlbChmYWN0b3IoVU4uU3ViLlJlZ2lvbikscmVmPSJDZW50cmFsIEFzaWEiKStkYXQkSERJX0NhdDIsIGRhdD1kYXQsIG1ldGhvZD0iTUwiKQpyb3VuZChleHAoY29lZihzdW1tYXJ5KG1vZGVsX1MpKVstMSxjKCJlc3RpbWF0ZSIsICJjaS5sYiIsICJjaS51YiIpXSksIDIpCnByaW50KG1vZGVsX1MsZGlnaXRzPTMpCgojIGFkZCBtYWxhcmlhIGJ1dCBzdWJzZXQgdG8gU1NBCm1vZGVsX1MgPC0gcm1hLmdsbW0oeGk9c3R1bnRfbiwgbmk9SGVpZ2h0X0FnZV9OLCBtZWFzdXJlPSJQTE8iLCBtb2RzID0gfiByZWxldmVsKGZhY3RvcihVTi5TdWIuUmVnaW9uKSxyZWY9IkNlbnRyYWwgQXNpYSIpK0hESV9DYXQyK3NjYWxlKE1hbF9QcmV2KSwgZGF0PWRhdCxzdWJzZXQgPSBTU0EgPT0gIlN1Yi1TYWhhcmFuIEFmcmljYSIsIG1ldGhvZD0iTUwiKQpyb3VuZChleHAoY29lZihzdW1tYXJ5KG1vZGVsX1MpKVstMSxjKCJlc3RpbWF0ZSIsICJjaS5sYiIsICJjaS51YiIpXSksIDIpCnByaW50KG1vZGVsX1MsZGlnaXRzPTMpCgpgYGAKCgpNZXRhLXJlZ3Jlc3Npb24uIEZpdHRpbmcgZ2VuZXJhbGl6ZWQgbGluZWFyIG1peGVkIGVmZmVjdHMgbW9kZWxzIGZvciB3YXN0aW5nCgpgYGB7ciB3YXN0aW5nIG1ldGEtcmVncmVzc2lvbn0KIyBVc2UgZ2xtbSB0byBlc3RpbWF0ZSB0aGUgcmlzayBvZiB3YXN0aW5nCm1vZGVsX1cgPC0gcm1hLmdsbW0oIHhpPXdhc3RlZF9uLCBuaT1XZWlnaHRfSGVpZ2h0X04sIG1lYXN1cmU9IlBMTyIsIG1vZHMgPSB+IHJlbGV2ZWwoZmFjdG9yKFVOLlN1Yi5SZWdpb24pLHJlZj0iQ2VudHJhbCBBc2lhIikrZGF0JEhESV9DYXQyLCBkYXQ9ZGF0LCBtZXRob2Q9Ik1MIikKcm91bmQoZXhwKGNvZWYoc3VtbWFyeShtb2RlbF9XKSlbLTEsYygiZXN0aW1hdGUiLCAiY2kubGIiLCAiY2kudWIiKV0pLCAyKQpwcmludChtb2RlbF9XLGRpZ2l0cz0zKQoKIyBhZGQgbWFsYXJpYSBidXQgc3Vic2V0IHRvIFNTQQptb2RlbF9XIDwtIHJtYS5nbG1tKHhpPXdhc3RlZF9uLCBuaT1XZWlnaHRfSGVpZ2h0X04sIG1lYXN1cmU9IlBMTyIsIG1vZHMgPSB+IHJlbGV2ZWwoZmFjdG9yKFVOLlN1Yi5SZWdpb24pLHJlZj0iQ2VudHJhbCBBc2lhIikrSERJX0NhdDIrc2NhbGUoTWFsX1ByZXYpLCBkYXQ9ZGF0LHN1YnNldCA9IFNTQSA9PSAiU3ViLVNhaGFyYW4gQWZyaWNhIiwgbWV0aG9kPSJNTCIpCnJvdW5kKGV4cChjb2VmKHN1bW1hcnkobW9kZWxfVykpWy0xLGMoImVzdGltYXRlIiwgImNpLmxiIiwgImNpLnViIildKSwgMikKcHJpbnQobW9kZWxfVyxkaWdpdHM9MykKCmBgYAoKTWV0YS1yZWdyZXNzaW9uLiBGaXR0aW5nIGdlbmVyYWxpemVkIGxpbmVhciBtaXhlZCBlZmZlY3RzIG1vZGVscyBmb3IgdW5kZXJ3ZWlnaHQKCmBgYHtyIHVuZGVyd2VpZ2h0IG1ldGEtcmVncmVzc2lvbn0KCiMgVXNlIGdsbW0gdG8gZXN0aW1hdGUgdGhlIHJpc2sgb2YgdW5kZXJ3ZWlnaHQKbW9kZWxfVSA8LSBybWEuZ2xtbSggeGk9dW5kZXJ3ZWlnaHRfbiwgbmk9V2VpZ2h0X0FnZV9OLCBtZWFzdXJlPSJQTE8iLCBtb2RzID0gfiByZWxldmVsKGZhY3RvcihVTi5TdWIuUmVnaW9uKSxyZWY9IkNlbnRyYWwgQXNpYSIpK2RhdCRIRElfQ2F0MiwgZGF0PWRhdCwgbWV0aG9kPSJNTCIpCnJvdW5kKGV4cChjb2VmKHN1bW1hcnkobW9kZWxfVSkpWy0xLGMoImVzdGltYXRlIiwgImNpLmxiIiwgImNpLnViIildKSwgMikKcHJpbnQobW9kZWxfVSxkaWdpdHM9MykKCiMgYWRkIG1hbGFyaWEgYnV0IHN1YnNldCB0byBTU0EKbW9kZWxfVSA8LSBybWEuZ2xtbSh4aT11bmRlcndlaWdodF9uLCBuaT1XZWlnaHRfQWdlX04sIG1lYXN1cmU9IlBMTyIsIG1vZHMgPSB+IHJlbGV2ZWwoZmFjdG9yKFVOLlN1Yi5SZWdpb24pLHJlZj0iQ2VudHJhbCBBc2lhIikrSERJX0NhdDIrc2NhbGUoTWFsX1ByZXYpLCBkYXQ9ZGF0LHN1YnNldCA9IFNTQSA9PSAiU3ViLVNhaGFyYW4gQWZyaWNhIiwgbWV0aG9kPSJNTCIpCnJvdW5kKGV4cChjb2VmKHN1bW1hcnkobW9kZWxfVSkpWy0xLGMoImVzdGltYXRlIiwgImNpLmxiIiwgImNpLnViIildKSwgMikKcHJpbnQobW9kZWxfVSxkaWdpdHM9MykKCmBgYAoKCgoKCgoKIyBwbG90IGZvciBIREk6IGxpbmVhciB0cmVuZCArIGNvbmZpZGVuY2UgaW50ZXJ2YWwKCmBgYHtyfQojIHBsb3Qgc3R1bnRpbmcgYXMgZnVuY3Rpb24gb2YgSERJCgpzMT1nZ3Bsb3QoZGF0LCBhZXMoeD1IRElfMjAxOCwgeT1zdHVudCkpICsKICBnZW9tX3BvaW50KGFscGhhPTAuOCwgc2l6ZT0zKSArCiAgZ2VvbV9zbW9vdGgobWV0aG9kPWxtICwgY29sb3I9InJlZCIsIGZpbGw9IiM2OWIzYTIiLCBzZT1UUlVFKSArCiAgdGhlbWVfaXBzdW0oKQoKczE9czErIHhsYWIoIkh1bWFuIGRldmVsb3BtZW50IGluZGV4IikrCiAgeWxhYignUHJldmFsZW5jZSBvZiBzdHVudGluZyAoJSknKSsKICB0aGVtZSgKICAgIGF4aXMudGl0bGUueD1lbGVtZW50X3RleHQoYW5nbGU9MCwgY29sb3I9J2JsYWNrJyxmYWNlID0gImJvbGQiLHNpemUgPSAyMCksCiAgICBheGlzLnRpdGxlLnk9ZWxlbWVudF90ZXh0KGFuZ2xlPTkwLCBjb2xvcj0nYmxhY2snLCBmYWNlPSdib2xkJywgc2l6ZT0yMCksCiAgICBheGlzLnRleHQueCA9IGVsZW1lbnRfdGV4dChmYWNlPSJib2xkIiwgY29sb3I9ImJsYWNrIixzaXplPTIwLCBhbmdsZT0wKSwKICAgIGF4aXMudGV4dC55ID0gZWxlbWVudF90ZXh0KGZhY2U9ImJvbGQiLCBjb2xvcj0iYmxhY2siLCBzaXplPTIwLCBhbmdsZT0wKSwKICAgIGxlZ2VuZC50ZXh0ID0gZWxlbWVudF90ZXh0KGNvbG91cj0iYmxhY2siLCBzaXplPTIwLGZhY2U9ImJvbGQiKSwKICAgIGxlZ2VuZC50aXRsZSA9IGVsZW1lbnRfdGV4dChjb2xvdXI9ImJsYWNrIiwgc2l6ZT0yMCxmYWNlPSJib2xkIikpCnMxCmNvci50ZXN0KGRhdCRIRElfMjAxOCwgZGF0JHN0dW50LCBtZXRob2Q9Yygic3BlYXJtYW4iKSkKCiMgcGxvdCB3YXN0aW5nCgp3MT1nZ3Bsb3QoZGF0LCBhZXMoeD1IRElfMjAxOCwgeT13YXN0ZWQpKSArCiAgZ2VvbV9wb2ludChhbHBoYT0wLjgsIHNpemU9MykgKwogIGdlb21fc21vb3RoKG1ldGhvZD1sbSAsIGNvbG9yPSJyZWQiLCBmaWxsPSIjNjliM2EyIiwgc2U9VFJVRSkgKwogIHRoZW1lX2lwc3VtKCkKCncxPXcxKyB4bGFiKCJIdW1hbiBkZXZlbG9wbWVudCBpbmRleCIpKwogIHlsYWIoJ1ByZXZhbGVuY2Ugb2Ygd2FzdGluZyAoJSknKSsKICB0aGVtZSgKICAgIGF4aXMudGl0bGUueD1lbGVtZW50X3RleHQoYW5nbGU9MCwgY29sb3I9J2JsYWNrJyxmYWNlID0gImJvbGQiLHNpemUgPSAyMCksCiAgICBheGlzLnRpdGxlLnk9ZWxlbWVudF90ZXh0KGFuZ2xlPTkwLCBjb2xvcj0nYmxhY2snLCBmYWNlPSdib2xkJywgc2l6ZT0yMCksCiAgICBheGlzLnRleHQueCA9IGVsZW1lbnRfdGV4dChmYWNlPSJib2xkIiwgY29sb3I9ImJsYWNrIixzaXplPTIwLCBhbmdsZT0wKSwKICAgIGF4aXMudGV4dC55ID0gZWxlbWVudF90ZXh0KGZhY2U9ImJvbGQiLCBjb2xvcj0iYmxhY2siLCBzaXplPTIwLCBhbmdsZT0wKSwKICAgIGxlZ2VuZC50ZXh0ID0gZWxlbWVudF90ZXh0KGNvbG91cj0iYmxhY2siLCBzaXplPTIwLGZhY2U9ImJvbGQiKSwKICAgIGxlZ2VuZC50aXRsZSA9IGVsZW1lbnRfdGV4dChjb2xvdXI9ImJsYWNrIiwgc2l6ZT0yMCxmYWNlPSJib2xkIikpCncxCmNvci50ZXN0KGRhdCRIRElfMjAxOCwgZGF0JHdhc3RlZCwgbWV0aG9kPWMoInNwZWFybWFuIikpCgojIHBsb3QgdW5kZXJ3ZWlnaHQKCnUxPWdncGxvdChkYXQsIGFlcyh4PUhESV8yMDE4LCB5PXVuZGVyd2VpZ2h0KSkgKwogIGdlb21fcG9pbnQoYWxwaGE9MC44LCBzaXplPTMpICsKICBnZW9tX3Ntb290aChtZXRob2Q9bG0gLCBjb2xvcj0icmVkIiwgZmlsbD0iIzY5YjNhMiIsIHNlPVRSVUUpICsKICB0aGVtZV9pcHN1bSgpCgp1MT11MSsgeGxhYigiSHVtYW4gZGV2ZWxvcG1lbnQgaW5kZXgiKSsKICB5bGFiKCdQcmV2YWxlbmNlIG9mIHVuZGVyd2VpZ2h0ICglKScpKwogIHRoZW1lKAogICAgYXhpcy50aXRsZS54PWVsZW1lbnRfdGV4dChhbmdsZT0wLCBjb2xvcj0nYmxhY2snLGZhY2UgPSAiYm9sZCIsc2l6ZSA9IDIwKSwKICAgIGF4aXMudGl0bGUueT1lbGVtZW50X3RleHQoYW5nbGU9OTAsIGNvbG9yPSdibGFjaycsIGZhY2U9J2JvbGQnLCBzaXplPTIwKSwKICAgIGF4aXMudGV4dC54ID0gZWxlbWVudF90ZXh0KGZhY2U9ImJvbGQiLCBjb2xvcj0iYmxhY2siLHNpemU9MjAsIGFuZ2xlPTApLAogICAgYXhpcy50ZXh0LnkgPSBlbGVtZW50X3RleHQoZmFjZT0iYm9sZCIsIGNvbG9yPSJibGFjayIsIHNpemU9MjAsIGFuZ2xlPTApLAogICAgbGVnZW5kLnRleHQgPSBlbGVtZW50X3RleHQoY29sb3VyPSJibGFjayIsIHNpemU9MjAsZmFjZT0iYm9sZCIpLAogICAgbGVnZW5kLnRpdGxlID0gZWxlbWVudF90ZXh0KGNvbG91cj0iYmxhY2siLCBzaXplPTIwLGZhY2U9ImJvbGQiKSkKdTEKCmNvci50ZXN0KGRhdCRIRElfMjAxOCwgZGF0JHVuZGVyd2VpZ2h0LCBtZXRob2Q9Yygic3BlYXJtYW4iKSkKCmBgYAoKIyBwbG90IGZvciBNYWxhcmlhOiBsaW5lYXIgdHJlbmQgKyBjb25maWRlbmNlIGludGVydmFsCgpgYGB7cn0KIyBwbG90IHN0dW50aW5nIGFzIGZ1bmN0aW9uIG9mIG1hbGFyaWEKCnMxPWdncGxvdChzdWJzZXQoZGF0LCBVTi5SZWdpb25zICVpbiUgYygiQWZyaWNhIikpLCBhZXMoeD1NYWxfUHJldiwgeT1zdHVudCkpICsKICBnZW9tX3BvaW50KGFscGhhPTAuOCwgc2l6ZT0zKSArCiAgZ2VvbV9zbW9vdGgobWV0aG9kPWxtICwgY29sb3I9InJlZCIsIGZpbGw9IiM2OWIzYTIiLCBzZT1UUlVFKSArCiAgdGhlbWVfaXBzdW0oKQoKCnMxPXMxKyB4bGFiKCJNYWxhcmlhIHByZXZhbGVuY2UgKCUpIikrCiAgeWxhYignUHJldmFsZW5jZSBvZiBzdHVudGluZyAoJSknKSsKICB0aGVtZSgKICAgIGF4aXMudGl0bGUueD1lbGVtZW50X3RleHQoYW5nbGU9MCwgY29sb3I9J2JsYWNrJyxmYWNlID0gImJvbGQiLHNpemUgPSAyMCksCiAgICBheGlzLnRpdGxlLnk9ZWxlbWVudF90ZXh0KGFuZ2xlPTkwLCBjb2xvcj0nYmxhY2snLCBmYWNlPSdib2xkJywgc2l6ZT0yMCksCiAgICBheGlzLnRleHQueCA9IGVsZW1lbnRfdGV4dChmYWNlPSJib2xkIiwgY29sb3I9ImJsYWNrIixzaXplPTIwLCBhbmdsZT0wKSwKICAgIGF4aXMudGV4dC55ID0gZWxlbWVudF90ZXh0KGZhY2U9ImJvbGQiLCBjb2xvcj0iYmxhY2siLCBzaXplPTIwLCBhbmdsZT0wKSwKICAgIGxlZ2VuZC50ZXh0ID0gZWxlbWVudF90ZXh0KGNvbG91cj0iYmxhY2siLCBzaXplPTIwLGZhY2U9ImJvbGQiKSwKICAgIGxlZ2VuZC50aXRsZSA9IGVsZW1lbnRfdGV4dChjb2xvdXI9ImJsYWNrIiwgc2l6ZT0yMCxmYWNlPSJib2xkIikpCnMxCmNvci50ZXN0KGRhdCRNYWwyLCBkYXQkc3R1bnQsIG1ldGhvZD1jKCJzcGVhcm1hbiIpKQoKIyBwbG90IHdhc3RpbmcKCncxPWdncGxvdChzdWJzZXQoZGF0LCBVTi5SZWdpb25zICVpbiUgYygiQWZyaWNhIikpLCBhZXMoeD1NYWxfUHJldiwgeT13YXN0ZWQpKSArCiAgZ2VvbV9wb2ludChhbHBoYT0wLjgsIHNpemU9MykgKwogIGdlb21fc21vb3RoKG1ldGhvZD1sbSAsIGNvbG9yPSJyZWQiLCBmaWxsPSIjNjliM2EyIiwgc2U9VFJVRSkgKwogIHRoZW1lX2lwc3VtKCkKCncxPXcxKyB4bGFiKCJNYWxhcmlhIHByZXZhbGVuY2UgKCUpIikrCiAgeWxhYignUHJldmFsZW5jZSBvZiB3YXN0aW5nICglKScpKwogIHRoZW1lKAogICAgYXhpcy50aXRsZS54PWVsZW1lbnRfdGV4dChhbmdsZT0wLCBjb2xvcj0nYmxhY2snLGZhY2UgPSAiYm9sZCIsc2l6ZSA9IDIwKSwKICAgIGF4aXMudGl0bGUueT1lbGVtZW50X3RleHQoYW5nbGU9OTAsIGNvbG9yPSdibGFjaycsIGZhY2U9J2JvbGQnLCBzaXplPTIwKSwKICAgIGF4aXMudGV4dC54ID0gZWxlbWVudF90ZXh0KGZhY2U9ImJvbGQiLCBjb2xvcj0iYmxhY2siLHNpemU9MjAsIGFuZ2xlPTApLAogICAgYXhpcy50ZXh0LnkgPSBlbGVtZW50X3RleHQoZmFjZT0iYm9sZCIsIGNvbG9yPSJibGFjayIsIHNpemU9MjAsIGFuZ2xlPTApLAogICAgbGVnZW5kLnRleHQgPSBlbGVtZW50X3RleHQoY29sb3VyPSJibGFjayIsIHNpemU9MjAsZmFjZT0iYm9sZCIpLAogICAgbGVnZW5kLnRpdGxlID0gZWxlbWVudF90ZXh0KGNvbG91cj0iYmxhY2siLCBzaXplPTIwLGZhY2U9ImJvbGQiKSkKdzEKCmNvci50ZXN0KGRhdCRNYWwyLCBkYXQkd2FzdGVkLCBtZXRob2Q9Yygic3BlYXJtYW4iKSkKIyBwbG90IHVuZGVyd2VpZ2h0Cgp1MT1nZ3Bsb3Qoc3Vic2V0KGRhdCwgVU4uUmVnaW9ucyAlaW4lIGMoIkFmcmljYSIpKSwgYWVzKHg9TWFsX1ByZXYsIHk9dW5kZXJ3ZWlnaHQpKSArCiAgZ2VvbV9wb2ludChhbHBoYT0wLjgsIHNpemU9MykgKwogIGdlb21fc21vb3RoKG1ldGhvZD1sbSAsIGNvbG9yPSJyZWQiLCBmaWxsPSIjNjliM2EyIiwgc2U9VFJVRSkgKwogIHRoZW1lX2lwc3VtKCkKCnUxPXUxKyB4bGFiKCJNYWxhcmlhIHByZXZhbGVuY2UgKCUpIikrCiAgeWxhYignUHJldmFsZW5jZSBvZiB1bmRlcndlaWdodCAoJSknKSsKICB0aGVtZSgKICAgIGF4aXMudGl0bGUueD1lbGVtZW50X3RleHQoYW5nbGU9MCwgY29sb3I9J2JsYWNrJyxmYWNlID0gImJvbGQiLHNpemUgPSAyMCksCiAgICBheGlzLnRpdGxlLnk9ZWxlbWVudF90ZXh0KGFuZ2xlPTkwLCBjb2xvcj0nYmxhY2snLCBmYWNlPSdib2xkJywgc2l6ZT0yMCksCiAgICBheGlzLnRleHQueCA9IGVsZW1lbnRfdGV4dChmYWNlPSJib2xkIiwgY29sb3I9ImJsYWNrIixzaXplPTIwLCBhbmdsZT0wKSwKICAgIGF4aXMudGV4dC55ID0gZWxlbWVudF90ZXh0KGZhY2U9ImJvbGQiLCBjb2xvcj0iYmxhY2siLCBzaXplPTIwLCBhbmdsZT0wKSwKICAgIGxlZ2VuZC50ZXh0ID0gZWxlbWVudF90ZXh0KGNvbG91cj0iYmxhY2siLCBzaXplPTIwLGZhY2U9ImJvbGQiKSwKICAgIGxlZ2VuZC50aXRsZSA9IGVsZW1lbnRfdGV4dChjb2xvdXI9ImJsYWNrIiwgc2l6ZT0yMCxmYWNlPSJib2xkIikpCnUxCgpjb3IudGVzdChkYXQkTWFsMiwgZGF0JHVuZGVyd2VpZ2h0LCBtZXRob2Q9Yygic3BlYXJtYW4iKSkKCmBgYAoKYGBge3J9CiMgVXNlIHJvdyBuYW1lcyBhcyBwb2ludCBsYWJlbHMKI2RmJG5hbWUgPC0gcm93bmFtZXMoZGYpCmVkPWdnc2NhdHRlcihkYXQsIHggPSAiSERJXzIwMTgiLCB5ID0gIlZhbHVlIiwgcGFsZXR0ZSA9ICJqY28iLCBhZGQgPSAicmVnLmxpbmUiLCBjb25mLmludCA9IFRSVUUsCiAgIGxhYmVsID0gIkNvdW50cnkiLCByZXBlbCA9IFRSVUUpKwogIHN0YXRfY29yKGFlcygpLCBtZXRob2QgPSAic3BlYXJtYW4iLCBsYWJlbC55ID0gOTApKwogIHRoZW1lX2lwc3VtKCkKCmVkPWVkKyB4bGFiKCJIdW1hbiBEZXZlbG9wbWVudCBJbmRleCIpKwogIHlsYWIoJ0VkdWNhdGlvbiBBdHRhaW5tZW50IGZvciBXb21lbicpKwogIHRoZW1lKAogICAgYXhpcy50aXRsZS54PWVsZW1lbnRfdGV4dChhbmdsZT0wLCBjb2xvcj0nYmxhY2snLGZhY2UgPSAiYm9sZCIsc2l6ZSA9IDE0KSwKICAgIGF4aXMudGl0bGUueT1lbGVtZW50X3RleHQoYW5nbGU9OTAsIGNvbG9yPSdibGFjaycsIGZhY2U9J2JvbGQnLCBzaXplPTE0KSwKICAgIGF4aXMudGV4dC54ID0gZWxlbWVudF90ZXh0KGZhY2U9ImJvbGQiLCBjb2xvcj0iYmxhY2siLHNpemU9MjAsIGFuZ2xlPTApLAogICAgYXhpcy50ZXh0LnkgPSBlbGVtZW50X3RleHQoZmFjZT0iYm9sZCIsIGNvbG9yPSJibGFjayIsIHNpemU9MjAsIGFuZ2xlPTApLAogICAgbGVnZW5kLnRleHQgPSBlbGVtZW50X3RleHQoY29sb3VyPSJibGFjayIsIHNpemU9MjAsZmFjZT0iYm9sZCIpLAogICAgbGVnZW5kLnRpdGxlID0gZWxlbWVudF90ZXh0KGNvbG91cj0iYmxhY2siLCBzaXplPTIwLGZhY2U9ImJvbGQiKSkKZWQKCmBgYAoK
