# Supplementary figures and images for: Global, regional and national epidemiology and prevalence of child stunting, wasting and underweight in low- and middle-income countries, 2006–2018
Source: Sci Rep. 2021 Mar 4;11:5204. doi: 10.1038/s41598-021-84302-w (PMC7933191; doi:10.1038/s41598-021-84302-w)

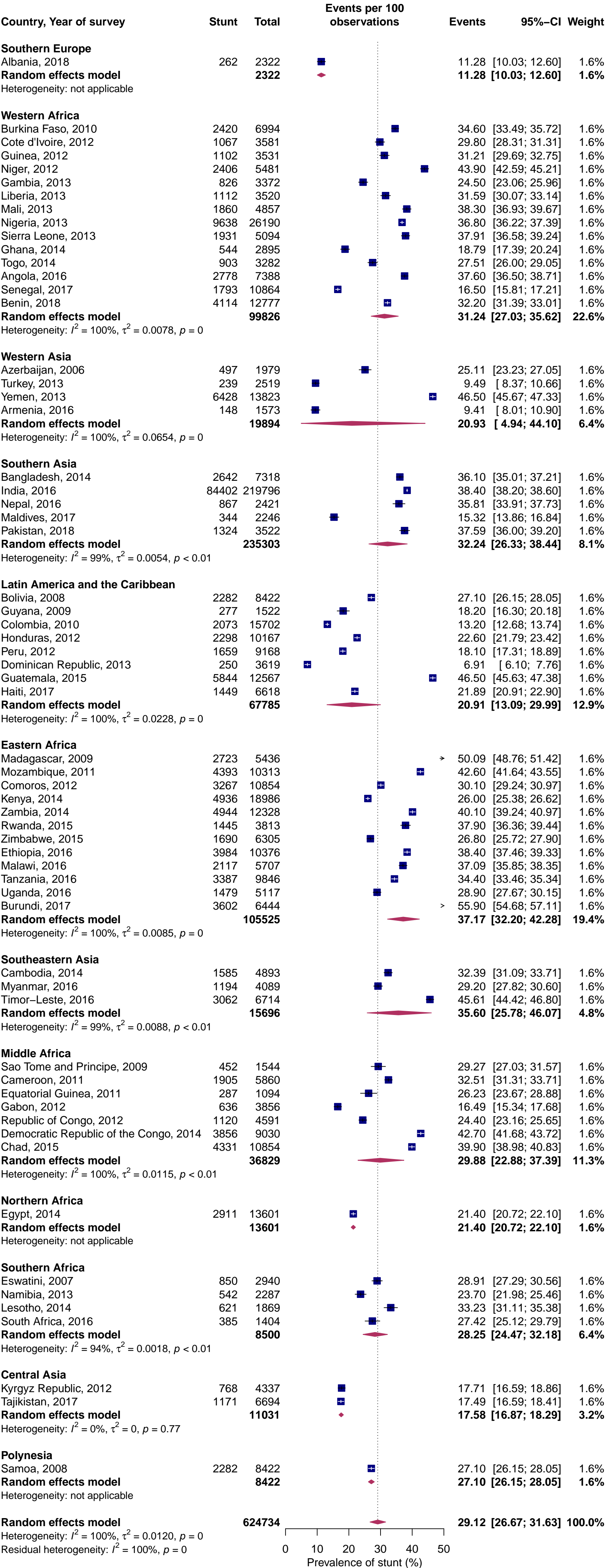

Supplement: Supplementary file 1 — Supplementary Information 1. [file 41598_2021_84302_MOESM1_ESM.zip › R code and Data/UNSunRegions.pdf]

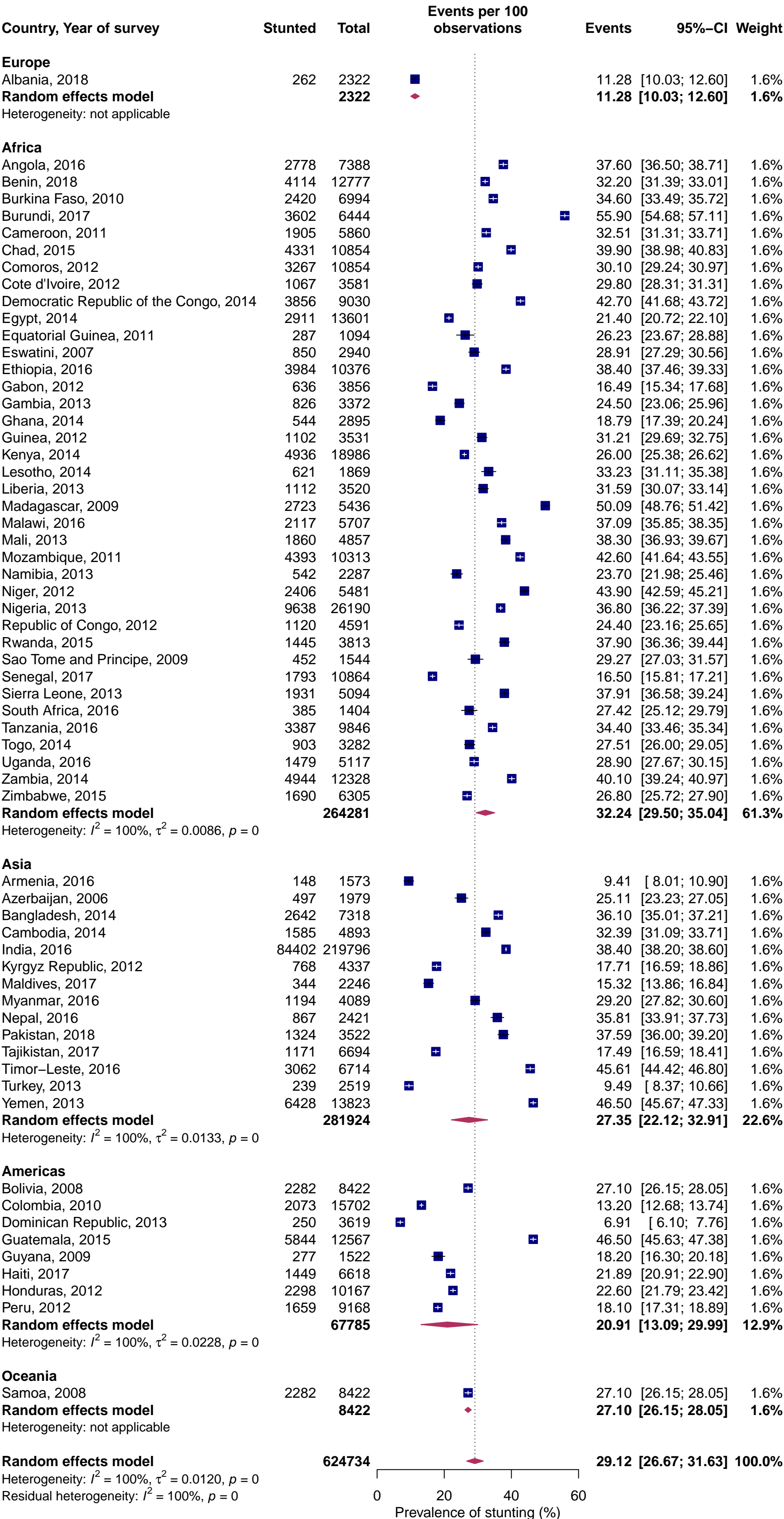

Supplement: Supplementary file 1 — Supplementary Information 1. [file 41598_2021_84302_MOESM1_ESM.zip › R code and Data/figs/SupplementaryFigureS5.pdf]

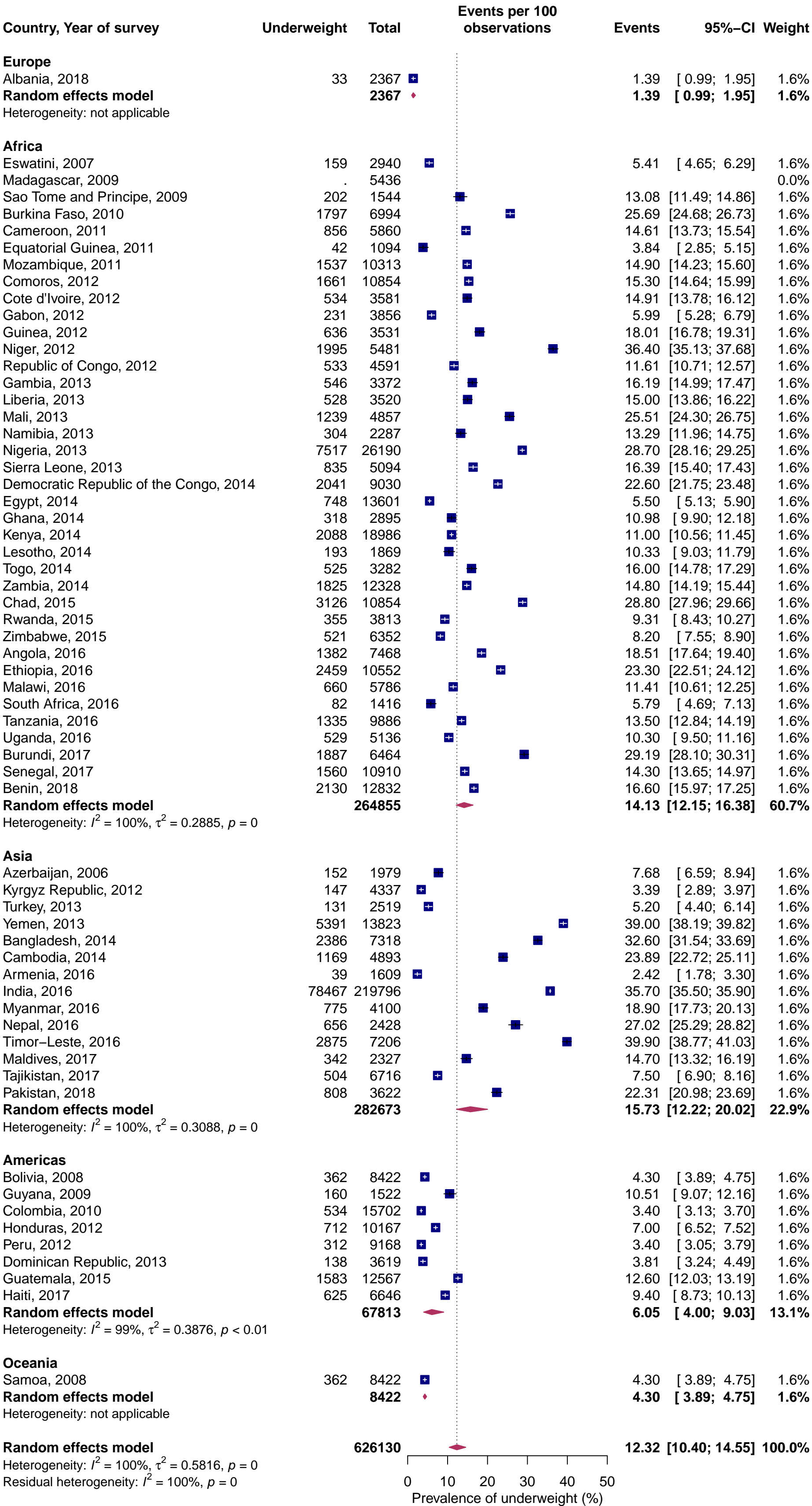

Supplement: Supplementary file 1 — Supplementary Information 1. [file 41598_2021_84302_MOESM1_ESM.zip › R code and Data/figs/SupplementaryFigureS7.pdf]

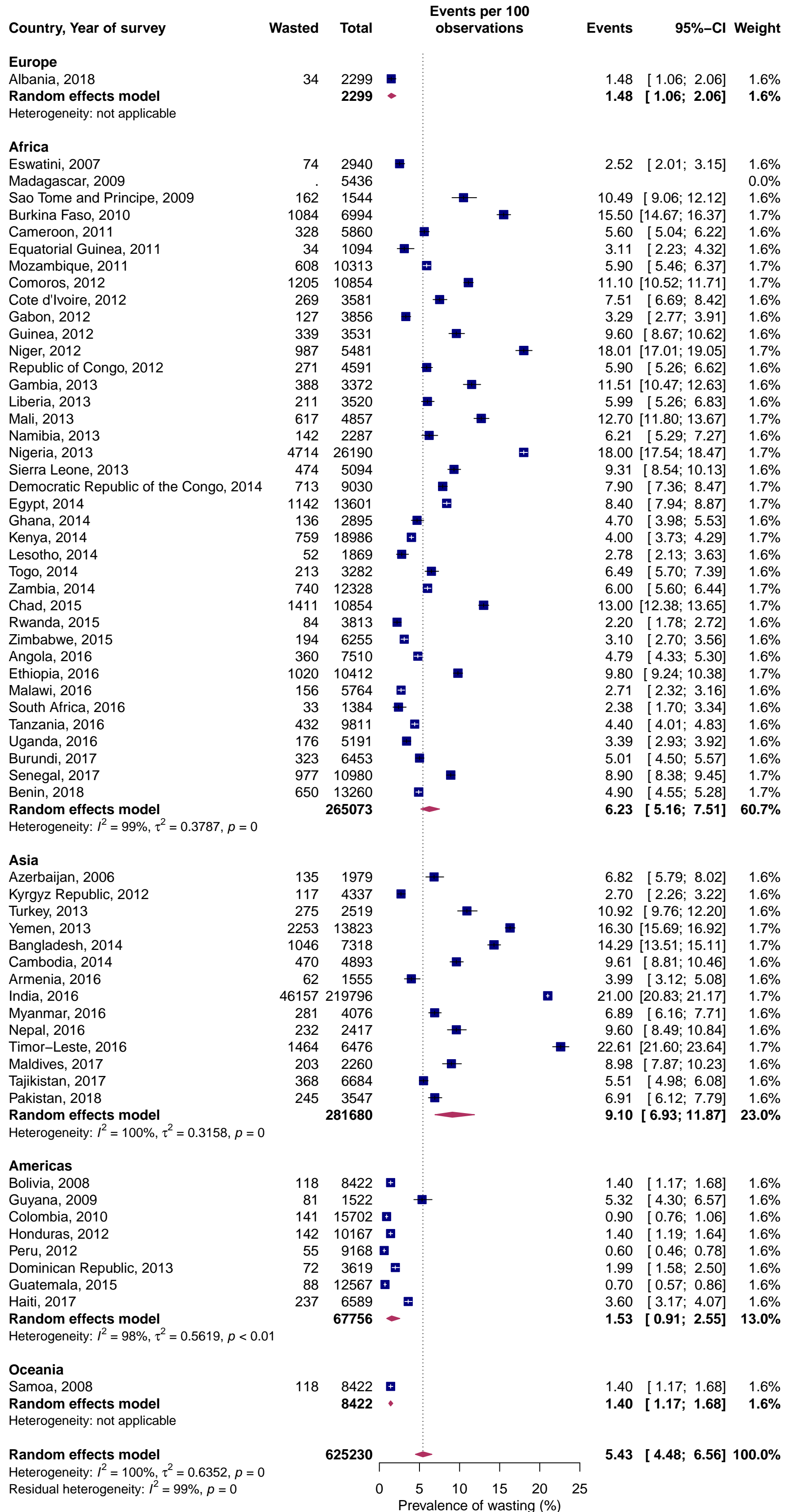

Supplement: Supplementary file 1 — Supplementary Information 1. [file 41598_2021_84302_MOESM1_ESM.zip › R code and Data/figs/SupplementaryFigureS6.pdf]

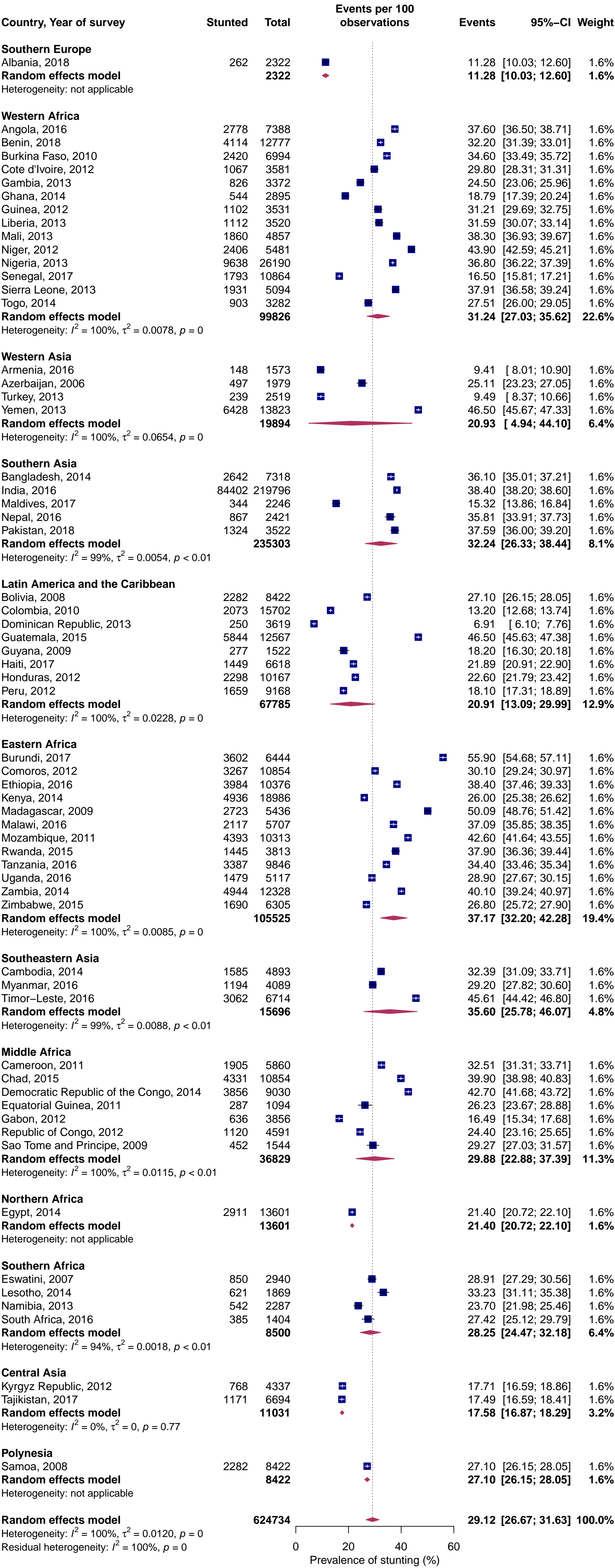

Supplement: Supplementary file 1 — Supplementary Information 1. [file 41598_2021_84302_MOESM1_ESM.zip › R code and Data/figs/Figure2.pdf]

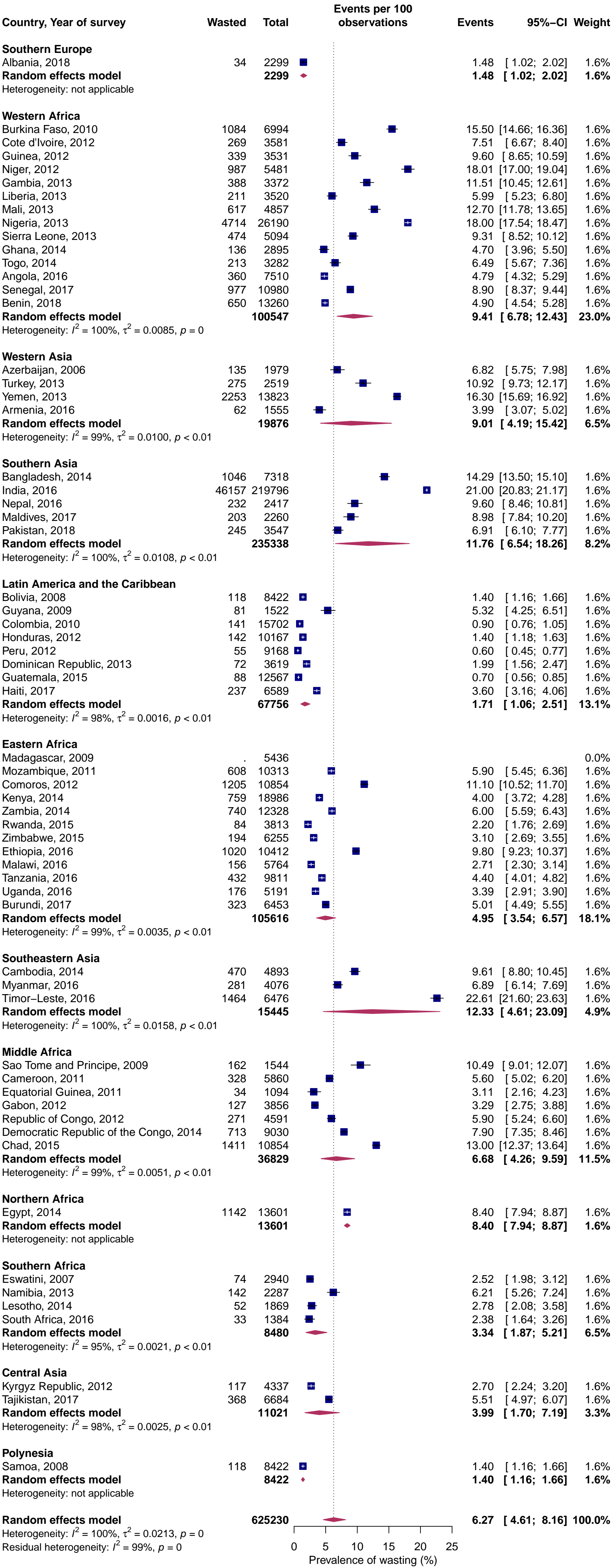

Supplement: Supplementary file 1 — Supplementary Information 1. [file 41598_2021_84302_MOESM1_ESM.zip › R code and Data/figs/Figure3.pdf]

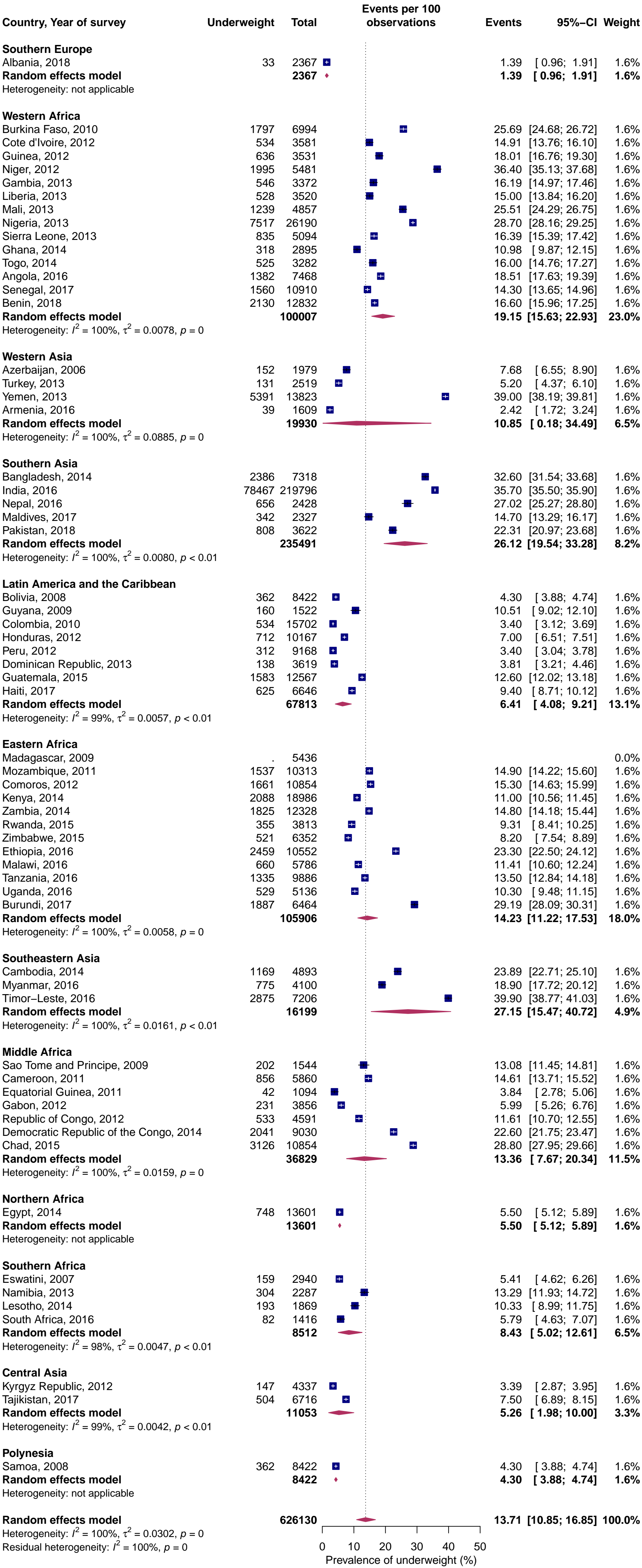

Supplement: Supplementary file 1 — Supplementary Information 1. [file 41598_2021_84302_MOESM1_ESM.zip › R code and Data/figs/Figure4.pdf]
