# Supplementary material for: Global, regional and national epidemiology and prevalence of child stunting, wasting and underweight in low- and middle-income countries, 2006–2018
Source: Sci Rep. 2021 Mar 4;11:5204. doi: 10.1038/s41598-021-84302-w (PMC7933191; doi:10.1038/s41598-021-84302-w)
Supplement: Supplementary file 2 — Supplementary Information 2. [file 41598_2021_84302_MOESM2_ESM.pdf]

Global, regional and national epidemiology and prevalence of child stunting, wasting and underweight in low- and middle- income countries, 2006-18

Paddy Ssentongo, MD, MPH[1,2,3] Anna E. Ssentongo, MPH[3,4], Djibril M. Ba, MPH[3,5] Jessica E. Ericson, MD, MPH[6], Muzi Na, PhD[7], Xiang Gao, MD, PhD[7], Claudio Fronterre[8], Vernon M. Chinchilli, PhD[3,] Steven J. Schiff, MD, PhD,[1,2,9,10,11]

[1]Center for Neural Engineering, The Pennsylvania State University, University Park, PA 16802, USA

[2]Department of Engineering Science and Mechanics, The Pennsylvania State University, University Park, PA 16802, USA

[3]Department of Public Health Sciences, The Pennsylvania State University College of Medicine, Hershey, PA 17033, USA

[4]Department of Surgery, The Pennsylvania State University College of Medicine, Hershey, PA 17033, USA

[5]Center for Applied Studies in Health Economics, The Pennsylvania State University College of Medicine, Hershey, PA 17033, USA

[6]Department of Pediatrics, The Pennsylvania State University College of Medicine, Hershey, PA 17033, USA

[7]Department of Nutritional Sciences, College of Health and Human Development, The Pennsylvania State University, University Park, PA 16802, USA

[8]Centre for Health Informatics, Computing, and Statistics, Lancaster University, Lancaster, United Kingdom

[9]The Center for Infectious Disease Dynamics, The Pennsylvania State University, University Park, PA 16802, USA

[10]Department of Neurosurgery, The Pennsylvania State University College of Medicine, Hershey, PA 17033, USA

[11]Department of Physics, The Pennsylvania State University, University Park, PA 16802, USA

Djibril M. Ba: djibrilba3@phs.psu.edu

Anna Ssentongo: assentongo@pennstatehealth.psu.edu

Jessica Ericson: jericson@pennstatehealth.psu.edu

Muzi Na: muzi.na@psu.edu

Xiang Gao: xxg14@psu.edu

Vernon M. Chinchilli: VChinchilli@pennstatehealth.psu.edu

Steven J. Schiff: steven.j.schiff22@gmail.com

To whom correspondence should be addressed:

Paddy Ssentongo, MD, MPH

500 University Drive

Penn State College of Medicine

Hershey PA, 17033 USA

814-777-2741

pssentongo@pennstatehealth.psu.edu

Country	Years of survey	Length/height-for-age N (% Stunted)	Weight-for-length/height N (%Wasted)	Weight-for-age N (% underweight)
**sub-Saharan Africa**				
Angola	2015-16	7388 (37.6)	7510 (4.8)	7468 (18.5)
Benin	2017-18	12777 (32.2)	13260 (4.9)	12832 (16.6)
Burkina Faso	2010	6994 (34.6)	6994 (15.5)	6994 (25.7)
Burundi	2016-17	6444 (55.9)	6453 (5)	6464 (29.2)
Cameroon	2011	5860 (32.5)	5860 (5.6)	5860 (14.6)
Chad	2014-15	10854 (39.9)	10854 (13)	10854 (28.8)
Comoros	2012	1804 (30.1)	1804 (11.1)	1804 (15.3)
Republic of Congo	2011-12	4591 (24.4)	4591 (5.9)	4591 (11.6)
Democratic Republic of the Congo	2013-14	9030 (42.7)	9030 (7.9)	9030 (22.6)
Equatorial Guinea	2011	1094 (26.2)	1094 (3.1)	1094 (3.8)
Ivory Coast	2011-12	3581 (29.8)	3581 (7.5)	3581 (14.9)
Swaziland	2006-07	2940 (28.9)	2940 (2.5)	2940 (5.4)
Ethiopia	2016	10376 (38.4)	10412 (9.8)	10552 (23.3)
Gabon	2012	3856 (16.5)	3856 (3.3)	3856 (6)
Gambia	2013	3372 (24.5)	3372 (11.5)	3372 (16.2)
Ghana	2014	2895 (18.8)	2895 (4.7)	2895 (11)
Guinea	2012	3531 (31.2)	3531 (9.6)	3531 (18)
Kenya	2014	18986 (26)	18986 (4)	18986 (11)
Lesotho	2014	1869 (33.2)	1869 (2.8)	1869 (10.3)
Liberia	2013	3520 (31.6)	3520 (6)	3520 (15)
Madagascar	2008-2009	5436 (50.1)	5436 (-)	5436 (-)
Malawi	2015-2016	5707 (37.1)	5764 (2.7)	5786 (11.4)
Mali	2012-13	4857 (38.3)	4857 (12.7)	4857 (25.5)
Mozambique	2011	10313 (42.6)	10313 (5.9)	10313 (14.9)
Namibia	2013	2287 (23.7)	2287 (6.2)	2287 (13.3)
Niger	2012	5481 (43.9)	5481 (18)	5481(36.4)
Nigeria	2013	26190 (36.8)	26190 (18)	26190)

Rwanda	2014-2015	3813 (37.9)	3813 (2.2)	3813 (9.3)
Sao Tome and Principe	2008-09	1544 (29.3)	1544 (10.5)	1544 (13.1)
Senegal	2017	10864 (16.5)	10980 (8.9)	10910 (14.3)
Sierra Leone	2013	5094 (37.9)	5094 (9.3)	5094 (16.4)
South Africa	2016	1404 (27.4)	1384 (2.4)	1416 (5.8)
Tanzania	2015-2016	9846 (34.4)	9811 (4.4)	9886 (13.5)
Togo	2013-14	3282 (27.5)	3282 (6.5)	3282 (16)
Uganda	2016	5117 (28.9)	5191 (3.4)	5136 (10.3)
Zambia	2013-2014	12328 (40.1)	12328 (6)	12328)
Zimbabwe	2015	6305 (26.8)	6255 (3.1)	6352 (8.2)
**North Africa**				
Egypt	2014	13601 (21.4)	13601 (8.4)	13601 (5.5)
**Southern Europe**				
Albania	2017-18	2322 (11.3)	2299 (1.5)	2367 (1.4)
**Western Asia**				
Armenia	2015-16	1573 (9.4)	1555 (4)	1609 (2.4)
Turkey	2013	2519 (9.5)	2519 (10.9)	2519 (5.2)
Yemen	2013	13823 (46.5)	13823 (16.3)	13823 (39)
Azerbaijan	2006	1979 (25.1)	1979 (6.8)	1979 (7.7)
**Central Asia**				
Kyrgyz Republic	2012	4337 (17.7)	4337 (2.7)	4337 (3.4)
Tajikistan	2017	6694 (17.5)	6684 (5.5)	6716 (7.5)
**East Asia/South Asia/Southeast Asia**				
Bangladesh	2014	7318 (36.1)	7318 (14.3)	7318 (32.6)
Cambodia	2014	4893 (32.4)	4893 (9.6)	4893 (23.9)
India	2015-16	219796 (38.4)	219796 (21)	219796 (35.7)
Maldives	2016-17	2246 (15.3)	2260 (9)	2327 (14.7)
Myanmar	2015-16	4089 (29.2)	4076 (6.9)	4100 (18.9)
Nepal	2016	2421 (35.8)	2417 (9.6)	2428 (27)
Pakistan	2017-18	3522 (37.6)	3547 (6.9)	3622 (22.3)
Timor-Leste	2016	6714 (45.6)	6476 (22.6)	7206 (39.9)
**Oceania**				
Samoa	2008	8422 (27.1)	8422 (1.4)	8422 (4.3)

**Latin America and Caribbean**				
Bolivia	2008	8422 (27.1)	8422 (1.4)	8422 (4.3)
Colombia	2010	15702 (13.2)	15702 (0.9)	15702 (3.4)
Dominican Republic	2013	3619 (6.9)	3619 (2)	3619 (3.8)
Guatemala	2014-15	12567 (46.5)	12567 (0.7)	12567 (12.6)
Guyana	2009	1522 (18.2)	1522 (5.3)	1522 (10.5)
Haiti	2016-17	6618 (21.9)	6589 (3.6)	6646 (9.4)
Honduras	2011-12	10167 (22.6)	10167 (1.4)	10167 (7)
Peru	2012	9168 (18.1)	9168 (0.6)	9168 (3.4)

Supplementary Table S1: Anthropometric measurements: Country-specific anthropometric indices showing number of children with anthropometric measurements.

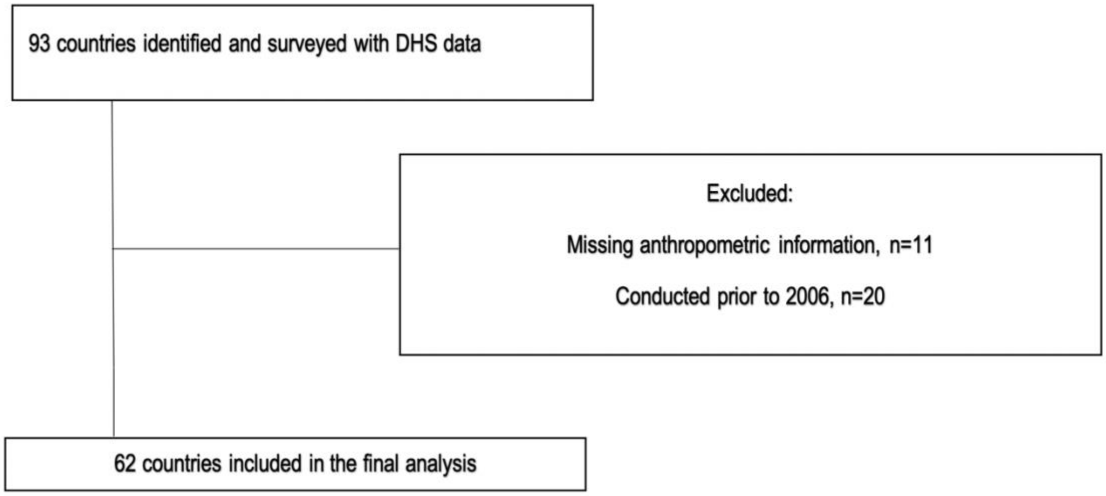

Supplementary Figure S1: Flow chart for country selection. In this study, nationally representative DHS data between 2006 and 2018 including anthropometric indices for each country was extracted. Surveys without anthropometric data were excluded from the analysis.

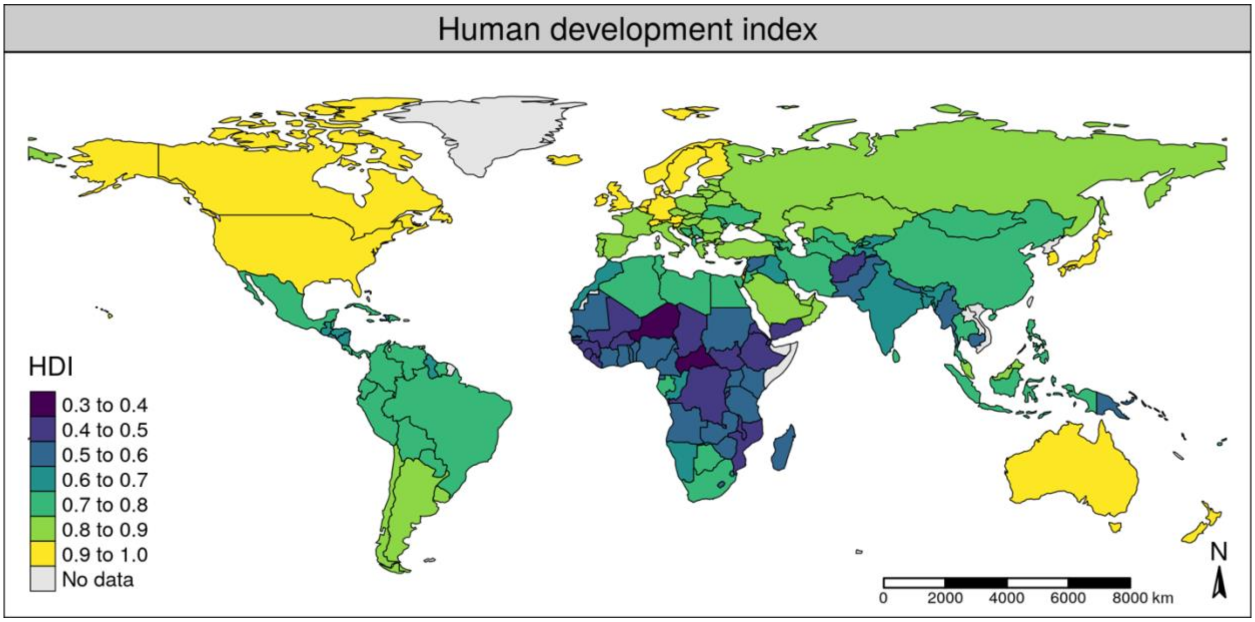

Supplemental Figure S2: Maps of covariates. Human development index data were extracted from the United Nations[1]. All the maps were produced with the R software for statistical computing version 3.6.3. The world shapefile was retrieved from the **spData** package (version 0.3.0, https://CRAN.R-project.org/package=spData) and the maps created with the **tmap** package (version 3.2.2, https://CRAN.R-project.org/package=tmap).

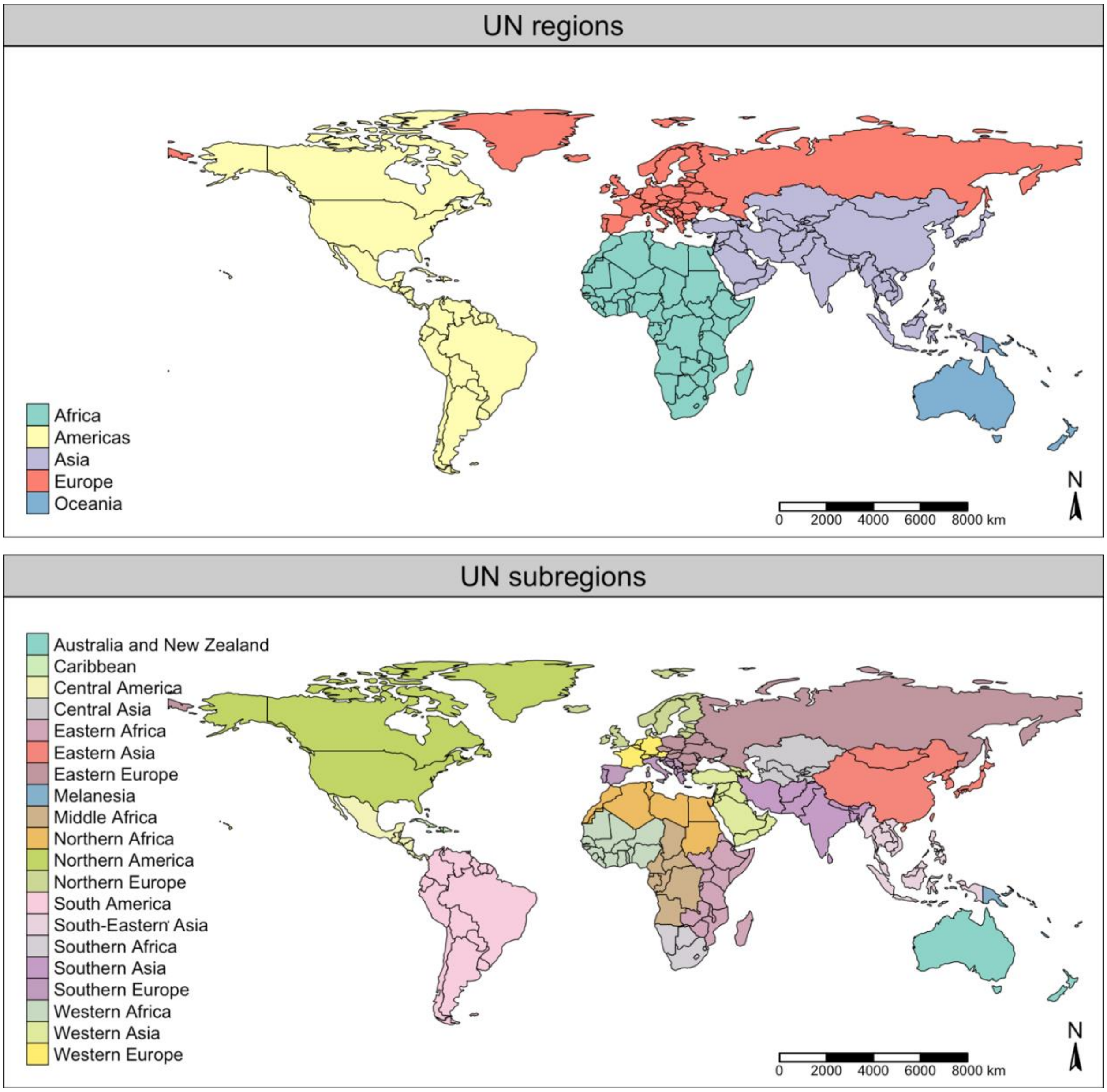

Supplemental Figure S3: Maps of covariates. UN regions (top panel) and Un subregions (bottom panel) used for aggregating data analysis in the current analysis[2]. All the maps were produced with the R software for statistical computing version 3.6.3. The world shapefile was retrieved from the **spData** package (version 0.3.0, https://CRAN.R-project.org/package=spData) and the maps created with the **tmap** package (version 3.2.2, https://CRAN.R-project.org/package=tmap).

Supplemental Figure S4: Forest plot of stunting prevalence by UN region of LMICs. Events values represent the number of cases of stunting expressed as a percentage. Blue squares and their corresponding lines are the point estimates and 95% confidence intervals (95% CI). Maroon diamonds represent the pooled estimate of the prevalence for each subgroup (width denotes 95% CI). Weights are from the random-effects analysis using the method of DerSimonian and Laird[3]. Heterogeneity by UN region: Africa (I^2 = 100%); Americas-Latin and Caribbean (I^2 = 100%); Europe (I^2 = not applicable); Asia (I^2 = 100%); and Oceania (I^2 = not applicable); p for interaction comparing the different subgroups < 0.0001.)

Country, Year of survey	Stunted	Total	Events	95%−CI	Weight
**Europe**					
Albania, 2018	262	2322	11.28	[10.03; 12.60]	1.6%
**Random effects model**		**2322**	**11.28**	**[10.03; 12.60]**	**1.6%**
Heterogeneity: not applicable					
**Africa**					
Angola, 2016	2778	7388	37.60	[36.50; 38.71]	1.6%
Benin, 2018	4114	12777	32.20	[31.39; 33.01]	1.6%
Burkina Faso, 2010	2420	6994	34.60	[33.49; 35.72]	1.6%
Burundi, 2017	3602	6444	55.90	[54.68; 57.11]	1.6%
Cameroon, 2011	1905	5860	32.51	[31.31; 33.71]	1.6%
Chad, 2015	4331	10854	39.90	[38.98; 40.83]	1.6%
Comoros, 2012	3267	10854	30.10	[29.24; 30.97]	1.6%
Cote d'Ivoire, 2012	1067	3581	29.80	[28.31; 31.31]	1.6%
Democratic Republic of the Congo, 2014	3856	9030	42.70	[41.68; 43.72]	1.6%
Egypt, 2014	2911	13601	21.40	[20.72; 22.10]	1.6%
Equatorial Guinea, 2011	287	1094	26.23	[23.67; 28.88]	1.6%
Eswatini, 2007	850	2940	28.91	[27.29; 30.56]	1.6%
Ethiopia, 2016	3984	10376	38.40	[37.46; 39.33]	1.6%
Gabon, 2012	636	3856	16.49	[15.34; 17.68]	1.6%
Gambia, 2013	826	3372	24.50	[23.06; 25.96]	1.6%
Ghana, 2014	544	2895	18.79	[17.39; 20.24]	1.6%
Guinea, 2012	1102	3531	31.21	[29.69; 32.75]	1.6%
Kenya, 2014	4936	18986	26.00	[25.38; 26.62]	1.6%
Lesotho, 2014	621	1869	33.23	[31.11; 35.38]	1.6%
Liberia, 2013	1112	3520	31.59	[30.07; 33.14]	1.6%
Madagascar, 2009	2723	5436	50.09	[48.76; 51.42]	1.6%
Malawi, 2016	2117	5707	37.09	[35.85; 38.35]	1.6%
Mali, 2013	1860	4857	38.30	[36.93; 39.67]	1.6%
Mozambique, 2011	4393	10313	42.60	[41.64; 43.55]	1.6%
Namibia, 2013	542	2287	23.70	[21.98; 25.46]	1.6%
Niger, 2012	2406	5481	43.90	[42.59; 45.21]	1.6%
Nigeria, 2013	9638	26190	36.80	[36.22; 37.39]	1.6%
Republic of Congo, 2012	1120	4591	24.40	[23.16; 25.65]	1.6%
Rwanda, 2015	1445	3813	37.90	[36.36; 39.44]	1.6%
Sao Tome and Principe, 2009	452	1544	29.27	[27.03; 31.57]	1.6%
Senegal, 2017	1793	10864	16.50	[15.81; 17.21]	1.6%
Sierra Leone, 2013	1931	5094	37.91	[36.58; 39.24]	1.6%
South Africa, 2016	385	1404	27.42	[25.12; 29.79]	1.6%
Tanzania, 2016	3387	9846	34.40	[33.46; 35.34]	1.6%
Togo, 2014	903	3282	27.51	[26.00; 29.05]	1.6%
Uganda, 2016	1479	5117	28.90	[27.67; 30.15]	1.6%
Zambia, 2014	4944	12328	40.10	[39.24; 40.97]	1.6%
Zimbabwe, 2015	1690	6305	26.80	[25.72; 27.90]	1.6%
**Random effects model**		**264281**	**32.24**	**[29.50; 35.04]**	**61.3%**
Heterogeneity: $I^2 = 100\%$, $\tau^2 = 0.0086$, $p = 0$					
**Asia**					
Armenia, 2016	148	1573	9.41	[8.01; 10.90]	1.6%
Azerbaijan, 2006	497	1979	25.11	[23.23; 27.05]	1.6%
Bangladesh, 2014	2642	7318	36.10	[35.01; 37.21]	1.6%
Cambodia, 2014	1585	4893	32.39	[31.09; 33.71]	1.6%
India, 2016	84402	219796	38.40	[38.20; 38.60]	1.6%
Kyrgyz Republic, 2012	768	4337	17.71	[16.59; 18.86]	1.6%
Maldives, 2017	344	2246	15.32	[13.86; 16.84]	1.6%
Myanmar, 2016	1194	4089	29.20	[27.82; 30.60]	1.6%
Nepal, 2016	867	2421	35.81	[33.91; 37.73]	1.6%
Pakistan, 2018	1324	3522	37.59	[36.00; 39.20]	1.6%
Tajikistan, 2017	1171	6694	17.49	[16.59; 18.41]	1.6%
Timor−Leste, 2016	3062	6714	45.61	[44.42; 46.80]	1.6%
Turkey, 2013	239	2519	9.49	[8.37; 10.66]	1.6%
Yemen, 2013	6428	13823	46.50	[45.67; 47.33]	1.6%
**Random effects model**		**281924**	**27.35**	**[22.12; 32.91]**	**22.6%**
Heterogeneity: $I^2 = 100\%$, $\tau^2 = 0.0133$, $p = 0$					
**Americas**					
Bolivia, 2008	2282	8422	27.10	[26.15; 28.05]	1.6%
Colombia, 2010	2073	15702	13.20	[12.68; 13.74]	1.6%
Dominican Republic, 2013	250	3619	6.91	[6.10; 7.76]	1.6%
Guatemala, 2015	5844	12567	46.50	[45.63; 47.38]	1.6%
Guyana, 2009	277	1522	18.20	[16.30; 20.18]	1.6%
Haiti, 2017	1449	6618	21.89	[20.91; 22.90]	1.6%
Honduras, 2012	2298	10167	22.60	[21.79; 23.42]	1.6%
Peru, 2012	1659	9168	18.10	[17.31; 18.89]	1.6%
**Random effects model**		**67785**	**20.91**	**[13.09; 29.99]**	**12.9%**
Heterogeneity: $I^2 = 100\%$, $\tau^2 = 0.0228$, $p = 0$					
**Oceania**					
Samoa, 2008	2282	8422	27.10	[26.15; 28.05]	1.6%
**Random effects model**		**8422**	**27.10**	**[26.15; 28.05]**	**1.6%**
Heterogeneity: not applicable					
**Random effects model**		**624734**	**29.12**	**[26.67; 31.63]**	**100.0%**
Heterogeneity: $I^2 = 100\%$, $\tau^2 = 0.0120$, $p = 0$					
Residual heterogeneity: $I^2 = 100\%$, $p = 0$					

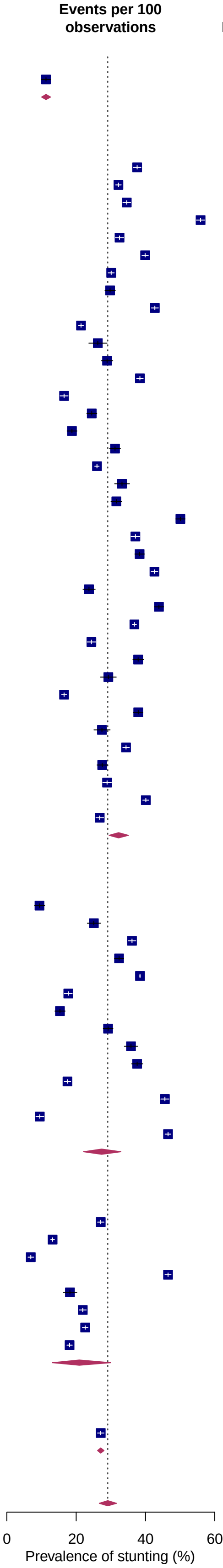

Supplemental Figure S5: Forest plot of wasting prevalence by UN region of LMICs. Events values represent the number of cases of wasting expressed as a percentage. Blue squares and their corresponding lines are the point estimates and 95% confidence intervals (95% CI). Maroon diamonds represent the pooled estimate of the prevalence for each subgroup (width denotes 95% CI). Weights are from the random-effects analysis using the method of DerSimonian and Laird[3]. Heterogeneity by UN region: Africa (I^2 = 99%); Americas-Latin and Caribbean (I^2 = 98%); Europe (I^2 = not applicable); Asia (I^2 = 100%); and Oceania (I^2 = not applicable); p for interaction comparing the different subgroups < 0.0001.)

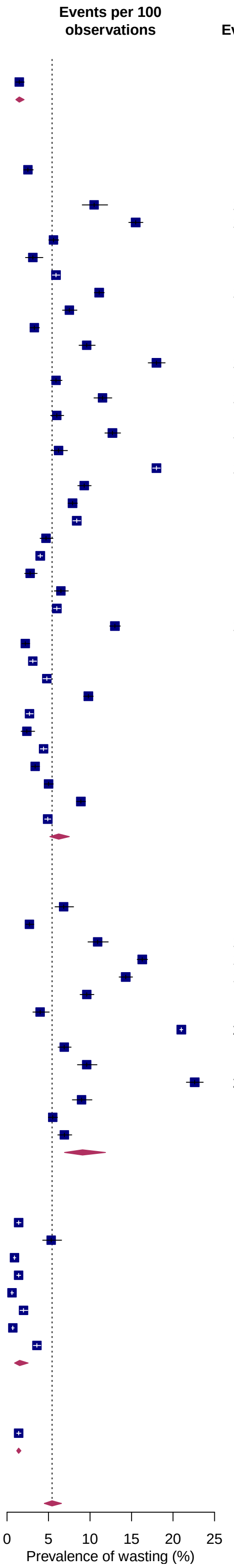

Country, Year of survey	Wasted	Total	Events	95%−CI	Weight
**Europe**					
Albania, 2018	34	2299	1.48	[1.06; 2.06]	1.6%
**Random effects model**		**2299**	**1.48**	**[1.06; 2.06]**	**1.6%**
Heterogeneity: not applicable					
**Africa**					
Eswatini, 2007	74	2940	2.52	[2.01; 3.15]	1.6%
Madagascar, 2009	.	5436			0.0%
Sao Tome and Principe, 2009	162	1544	10.49	[9.06; 12.12]	1.6%
Burkina Faso, 2010	1084	6994	15.50	[14.67; 16.37]	1.7%
Cameroon, 2011	328	5860	5.60	[5.04; 6.22]	1.6%
Equatorial Guinea, 2011	34	1094	3.11	[2.23; 4.32]	1.6%
Mozambique, 2011	608	10313	5.90	[5.46; 6.37]	1.7%
Comoros, 2012	1205	10854	11.10	[10.52; 11.71]	1.7%
Cote d'Ivoire, 2012	269	3581	7.51	[6.69; 8.42]	1.6%
Gabon, 2012	127	3856	3.29	[2.77; 3.91]	1.6%
Guinea, 2012	339	3531	9.60	[8.67; 10.62]	1.6%
Niger, 2012	987	5481	18.01	[17.01; 19.05]	1.7%
Republic of Congo, 2012	271	4591	5.90	[5.26; 6.62]	1.6%
Gambia, 2013	388	3372	11.51	[10.47; 12.63]	1.6%
Liberia, 2013	211	3520	5.99	[5.26; 6.83]	1.6%
Mali, 2013	617	4857	12.70	[11.80; 13.67]	1.7%
Namibia, 2013	142	2287	6.21	[5.29; 7.27]	1.6%
Nigeria, 2013	4714	26190	18.00	[17.54; 18.47]	1.7%
Sierra Leone, 2013	474	5094	9.31	[8.54; 10.13]	1.6%
Democratic Republic of the Congo, 2014	713	9030	7.90	[7.36; 8.47]	1.7%
Egypt, 2014	1142	13601	8.40	[7.94; 8.87]	1.7%
Ghana, 2014	136	2895	4.70	[3.98; 5.53]	1.6%
Kenya, 2014	759	18986	4.00	[3.73; 4.29]	1.7%
Lesotho, 2014	52	1869	2.78	[2.13; 3.63]	1.6%
Togo, 2014	213	3282	6.49	[5.70; 7.39]	1.6%
Zambia, 2014	740	12328	6.00	[5.60; 6.44]	1.7%
Chad, 2015	1411	10854	13.00	[12.38; 13.65]	1.7%
Rwanda, 2015	84	3813	2.20	[1.78; 2.72]	1.6%
Zimbabwe, 2015	194	6255	3.10	[2.70; 3.56]	1.6%
Angola, 2016	360	7510	4.79	[4.33; 5.30]	1.6%
Ethiopia, 2016	1020	10412	9.80	[9.24; 10.38]	1.7%
Malawi, 2016	156	5764	2.71	[2.32; 3.16]	1.6%
South Africa, 2016	33	1384	2.38	[1.70; 3.34]	1.6%
Tanzania, 2016	432	9811	4.40	[4.01; 4.83]	1.6%
Uganda, 2016	176	5191	3.39	[2.93; 3.92]	1.6%
Burundi, 2017	323	6453	5.01	[4.50; 5.57]	1.6%
Senegal, 2017	977	10980	8.90	[8.38; 9.45]	1.7%
Benin, 2018	650	13260	4.90	[4.55; 5.28]	1.7%
**Random effects model**		**265073**	**6.23**	**[5.16; 7.51]**	**60.7%**
Heterogeneity: $I^2 = 99\%$, $\tau^2 = 0.3787$, $p = 0$					
**Asia**					
Azerbaijan, 2006	135	1979	6.82	[5.79; 8.02]	1.6%
Kyrgyz Republic, 2012	117	4337	2.70	[2.26; 3.22]	1.6%
Turkey, 2013	275	2519	10.92	[9.76; 12.20]	1.6%
Yemen, 2013	2253	13823	16.30	[15.69; 16.92]	1.7%
Bangladesh, 2014	1046	7318	14.29	[13.51; 15.11]	1.7%
Cambodia, 2014	470	4893	9.61	[8.81; 10.46]	1.6%
Armenia, 2016	62	1555	3.99	[3.12; 5.08]	1.6%
India, 2016	46157	219796	21.00	[20.83; 21.17]	1.7%
Myanmar, 2016	281	4076	6.89	[6.16; 7.71]	1.6%
Nepal, 2016	232	2417	9.60	[8.49; 10.84]	1.6%
Timor−Leste, 2016	1464	6476	22.61	[21.60; 23.64]	1.7%
Maldives, 2017	203	2260	8.98	[7.87; 10.23]	1.6%
Tajikistan, 2017	368	6684	5.51	[4.98; 6.08]	1.6%
Pakistan, 2018	245	3547	6.91	[6.12; 7.79]	1.6%
**Random effects model**		**281680**	**9.10**	**[6.93; 11.87]**	**23.0%**
Heterogeneity: $I^2 = 100\%$, $\tau^2 = 0.3158$, $p = 0$					
**Americas**					
Bolivia, 2008	118	8422	1.40	[1.17; 1.68]	1.6%
Guyana, 2009	81	1522	5.32	[4.30; 6.57]	1.6%
Colombia, 2010	141	15702	0.90	[0.76; 1.06]	1.6%
Honduras, 2012	142	10167	1.40	[1.19; 1.64]	1.6%
Peru, 2012	55	9168	0.60	[0.46; 0.78]	1.6%
Dominican Republic, 2013	72	3619	1.99	[1.58; 2.50]	1.6%
Guatemala, 2015	88	12567	0.70	[0.57; 0.86]	1.6%
Haiti, 2017	237	6589	3.60	[3.17; 4.07]	1.6%
**Random effects model**		**67756**	**1.53**	**[0.91; 2.55]**	**13.0%**
Heterogeneity: $I^2 = 98\%$, $\tau^2 = 0.5619$, $p < 0.01$					
**Oceania**					
Samoa, 2008	118	8422	1.40	[1.17; 1.68]	1.6%
**Random effects model**		**8422**	**1.40**	**[1.17; 1.68]**	**1.6%**
Heterogeneity: not applicable					
**Random effects model**		**625230**	**5.43**	**[4.48; 6.56]**	**100.0%**
Heterogeneity: $I^2 = 100\%$, $\tau^2 = 0.6352$, $p = 0$					
Residual heterogeneity: $I^2 = 99\%$, $p = 0$					

Supplemental Figure S6: Forest plot of underweight prevalence by UN region of LMICs. Events values represent the number of cases of underweight expressed as a percentage. Blue squares and their corresponding lines are the point estimates and 95% confidence intervals (95% CI). Maroon diamonds represent the pooled estimate of the prevalence for each subgroup (width denotes 95% CI). Weights are from the random-effects analysis using the method of DerSimonian and Laird[3]. Heterogeneity by UN region: Africa (I^2 = 100%); Americas-Latin and Caribbean (I^2 = 99%); Europe (I^2 = not applicable); Asia (I^2 = 100%); and Oceania (I^2 = not applicable); p for interaction comparing the different subgroups < 0.0001.)

Country, Year of survey	Underweight	Total	Events per 100 observations	Events	95%−CI	Weight
**Europe**						
Albania, 2018	33	2367		1.39	[0.99; 1.95]	1.6%
**Random effects model**		**2367**		**1.39**	**[0.99; 1.95]**	**1.6%**
Heterogeneity: not applicable						
**Africa**						
Eswatini, 2007	159	2940		5.41	[4.65; 6.29]	1.6%
Madagascar, 2009	.	5436				0.0%
Sao Tome and Principe, 2009	202	1544		13.08	[11.49; 14.86]	1.6%
Burkina Faso, 2010	1797	6994		25.69	[24.68; 26.73]	1.6%
Cameroon, 2011	856	5860		14.61	[13.73; 15.54]	1.6%
Equatorial Guinea, 2011	42	1094		3.84	[2.85; 5.15]	1.6%
Mozambique, 2011	1537	10313		14.90	[14.23; 15.60]	1.6%
Comoros, 2012	1661	10854		15.30	[14.64; 15.99]	1.6%
Cote d'Ivoire, 2012	534	3581		14.91	[13.78; 16.12]	1.6%
Gabon, 2012	231	3856		5.99	[5.28; 6.79]	1.6%
Guinea, 2012	636	3531		18.01	[16.78; 19.31]	1.6%
Niger, 2012	1995	5481		36.40	[35.13; 37.68]	1.6%
Republic of Congo, 2012	533	4591		11.61	[10.71; 12.57]	1.6%
Gambia, 2013	546	3372		16.19	[14.99; 17.47]	1.6%
Liberia, 2013	528	3520		15.00	[13.86; 16.22]	1.6%
Mali, 2013	1239	4857		25.51	[24.30; 26.75]	1.6%
Namibia, 2013	304	2287		13.29	[11.96; 14.75]	1.6%
Nigeria, 2013	7517	26190		28.70	[28.16; 29.25]	1.6%
Sierra Leone, 2013	835	5094		16.39	[15.40; 17.43]	1.6%
Democratic Republic of the Congo, 2014	2041	9030		22.60	[21.75; 23.48]	1.6%
Egypt, 2014	748	13601		5.50	[5.13; 5.90]	1.6%
Ghana, 2014	318	2895		10.98	[9.90; 12.18]	1.6%
Kenya, 2014	2088	18986		11.00	[10.56; 11.45]	1.6%
Lesotho, 2014	193	1869		10.33	[9.03; 11.79]	1.6%
Togo, 2014	525	3282		16.00	[14.78; 17.29]	1.6%
Zambia, 2014	1825	12328		14.80	[14.19; 15.44]	1.6%
Chad, 2015	3126	10854		28.80	[27.96; 29.66]	1.6%
Rwanda, 2015	355	3813		9.31	[8.43; 10.27]	1.6%
Zimbabwe, 2015	521	6352		8.20	[7.55; 8.90]	1.6%
Angola, 2016	1382	7468		18.51	[17.64; 19.40]	1.6%
Ethiopia, 2016	2459	10552		23.30	[22.51; 24.12]	1.6%
Malawi, 2016	660	5786		11.41	[10.61; 12.25]	1.6%
South Africa, 2016	82	1416		5.79	[4.69; 7.13]	1.6%
Tanzania, 2016	1335	9886		13.50	[12.84; 14.19]	1.6%
Uganda, 2016	529	5136		10.30	[9.50; 11.16]	1.6%
Burundi, 2017	1887	6464		29.19	[28.10; 30.31]	1.6%
Senegal, 2017	1560	10910		14.30	[13.65; 14.97]	1.6%
Benin, 2018	2130	12832		16.60	[15.97; 17.25]	1.6%
**Random effects model**		**264855**		**14.13**	**[12.15; 16.38]**	**60.7%**
Heterogeneity: I^2 = 100%, τ^2 = 0.2885, p = 0						
**Asia**						
Azerbaijan, 2006	152	1979		7.68	[6.59; 8.94]	1.6%
Kyrgyz Republic, 2012	147	4337		3.39	[2.89; 3.97]	1.6%
Turkey, 2013	131	2519		5.20	[4.40; 6.14]	1.6%
Yemen, 2013	5391	13823		39.00	[38.19; 39.82]	1.6%
Bangladesh, 2014	2386	7318		32.60	[31.54; 33.69]	1.6%
Cambodia, 2014	1169	4893		23.89	[22.72; 25.11]	1.6%
Armenia, 2016	39	1609		2.42	[1.78; 3.30]	1.6%
India, 2016	78467	219796		35.70	[35.50; 35.90]	1.6%
Myanmar, 2016	775	4100		18.90	[17.73; 20.13]	1.6%
Nepal, 2016	656	2428		27.02	[25.29; 28.82]	1.6%
Timor−Leste, 2016	2875	7206		39.90	[38.77; 41.03]	1.6%
Maldives, 2017	342	2327		14.70	[13.32; 16.19]	1.6%
Tajikistan, 2017	504	6716		7.50	[6.90; 8.16]	1.6%
Pakistan, 2018	808	3622		22.31	[20.98; 23.69]	1.6%
**Random effects model**		**282673**		**15.73**	**[12.22; 20.02]**	**22.9%**
Heterogeneity: I^2 = 100%, τ^2 = 0.3088, p = 0						
**Americas**						
Bolivia, 2008	362	8422		4.30	[3.89; 4.75]	1.6%
Guyana, 2009	160	1522		10.51	[9.07; 12.16]	1.6%
Colombia, 2010	534	15702		3.40	[3.13; 3.70]	1.6%
Honduras, 2012	712	10167		7.00	[6.52; 7.52]	1.6%
Peru, 2012	312	9168		3.40	[3.05; 3.79]	1.6%
Dominican Republic, 2013	138	3619		3.81	[3.24; 4.49]	1.6%
Guatemala, 2015	1583	12567		12.60	[12.03; 13.19]	1.6%
Haiti, 2017	625	6646		9.40	[8.73; 10.13]	1.6%
**Random effects model**		**67813**		**6.05**	**[4.00; 9.03]**	**13.1%**
Heterogeneity: I^2 = 99%, τ^2 = 0.3876, p < 0.01						
**Oceania**						
Samoa, 2008	362	8422		4.30	[3.89; 4.75]	1.6%
**Random effects model**		**8422**		**4.30**	**[3.89; 4.75]**	**1.6%**
Heterogeneity: not applicable						
**Random effects model**		**626130**		**12.32**	**[10.40; 14.55]**	**100.0%**
Heterogeneity: I^2 = 100%, τ^2 = 0.5816, p = 0						
Residual heterogeneity: I^2 = 100%, p = 0						

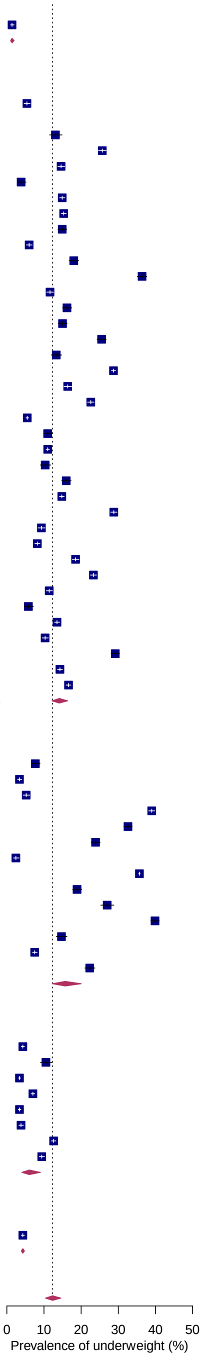

Equation for random-effects analysis using the method of DerSimonian and Laird[3]

Suppose that there are K studies/surveys. The estimated treatment effect for a binary response variable (logarithm of the odds ratio) in the kth study, $k = 1,2, \dots, K$, is Y_k. The estimated variance of Y_k in the kth study is S_k^2. The weight for the estimated treatment effect in the kth study in the fixed-effects model is $w_k = 1/S_k^2$. The overall weighted treatment effect in the mixed-effects model is

$$Y_k = \theta + u_k + e_k$$

where

1. Y_k is the observed effect in the kth study
2. θ is the pooled population parameter of interest (natural logarithm of the population odds ratio)
3. e_k is the random error term for the kth study
4. u_k is a random effect for the kth study, $k = 1,2, \dots, K$.

It is assumed that $e_1, e_2, \dots, e_K$ are independent random variables with $e_k \sim N(0, \sigma_k^2), k = 1,2, \dots, K$. The variance term σ_k^2 reflects intra-study variability and its estimate is S_k^2. Usually, Y_k and S_k^2 (or S_k) are provided as descriptive statistics in the kth study report. The overall weighted treatment effect in the mixed -effects model is

$$Y = \left(\sum_{k=1}^{K} w_k Y_k\right) / \left(\sum_{k=1}^{K} w_k\right)$$

where $w_k = 1/\left(S_k^2 + \widehat{\omega}^2\right), k = 1,2, \dots, K$.

the variance of Y in the random-effects model is

$$S^2 = 1 \Bigg/ \left(\sum_{k=1}^{K} w_k\right)$$

The statistic for testing H_0: $study\ homogeneity$ is

$$Q = \sum_{k=1}^{K} w_k (Y_k - Y)^2 \sim \chi_{K-1}^2$$

References

1 United Nations Development Programme. Human development index (HDI). doi:http://hdr.undp.org/en/data (2020).

2 United Nations. *Definition of Regions*, <https://population.un.org/wpp/DefinitionOfRegions/> (2019).

3 DerSimonian, R. & Laird, N. Meta-analysis in clinical trials. *Controlled Clinical Trials* **7**, 177-188, doi:https://doi.org/10.1016/0197-2456(86)90046-2 (1986).
